# Mitoribosome insufficiency in β cells is associated with type 2 diabetes-like islet failure

**DOI:** 10.1038/s12276-022-00797-x

**Published:** 2022-07-08

**Authors:** Hyun Jung Hong, Kyong Hye Joung, Yong Kyung Kim, Min Jeong Choi, Seul Gi Kang, Jung Tae Kim, Yea Eun Kang, Joon Young Chang, Joon Ho Moon, Sangmi Jun, Hyun-Joo Ro, Yujeong Lee, Hyeongseok Kim, Jae-Hyung Park, Baeki E. Kang, Yunju Jo, Heejung Choi, Dongryeol Ryu, Chul-Ho Lee, Hail Kim, Kyu-Sang Park, Hyun Jin Kim, Minho Shong

**Affiliations:** 1 Research Center for Endocrine and Metabolic Diseases, Chungnam National University School of Medicine, Daejeon, 35015 Korea; 2 Department of Medical Science, Chungnam National University School of Medicine, Daejeon, 35015 Korea; 3 Department of Internal Medicine, Chungnam National University School of Medicine, Daejeon, 35015 Korea; 4 Graduate School of Medical Science and Engineering, Korea Advanced Institute of Science and Technology, Daejeon, 34141 Korea; 5 Center for Research Equipment, Korea Basic Science Institute, Cheongju, 28119 Korea; 6 Convergent Research Center for Emerging Virus Infection, Korea Research Institute of Chemical Technology, Daejeon, 34114 Korea; 7 Department of Biochemistry, Chungnam National University School of Medicine, Daejeon, 35015 Korea; 8 Department of Physiology, Keimyung University School of Medicine, Daegu, 704-200 Korea; 9 Department of Molecular Cell Biology, Sungkyunkwan University School of Medicine, Suwon, 16419 Korea; 10 Biomedical Institute for Convergence at SKKU (BICS), Sungkyunkwan University, Suwon, 16419 Korea; 11 Samsung Biomedical Research Institute, Samsung Medical Center, Seoul, 06351 Korea; 12 Animal Model Center, Korea Research Institute of Bioscience and Biotechnology, Daejeon, 34141 Korea; 13 Department of Physiology, Yonsei University Wonju College of Medicine, Wonju, 26426 Korea

**Keywords:** Type 2 diabetes, Pre-diabetes, Endocrine system and metabolic diseases

## Abstract

Genetic variations in mitoribosomal subunits and mitochondrial transcription factors are related to type 2 diabetes. However, the role of islet mitoribosomes in the development of type 2 diabetes has not been determined. We investigated the effects of the mitoribosomal gene on β-cell function and glucose homeostasis. Mitoribosomal gene expression was analyzed in datasets from the NCBI GEO website (GSE25724, GSE76894, and GSE76895) and the European Nucleotide Archive (ERP017126), which contain the transcriptomes of type 2 diabetic and nondiabetic organ donors. We found deregulation of most mitoribosomal genes in islets from individuals with type 2 diabetes, including partial downregulation of CRIF1. The phenotypes of haploinsufficiency in a single mitoribosomal gene were examined using β-cell-specific *Crif1* (*Mrpl59*) heterozygous-deficient mice. *Crif1*^*beta+/−*^ mice had normal glucose tolerance, but their islets showed a loss of first-phase glucose-stimulated insulin secretion. They also showed increased β-cell mass associated with higher expression of *Reg* family genes. However, *Crif1*^*beta+/−*^ mice showed earlier islet failure in response to high-fat feeding, which was exacerbated by aging. Haploinsufficiency of a single mitoribosomal gene predisposes rodents to glucose intolerance, which resembles the early stages of type 2 diabetes in humans.

## Diabetes: A mitochondrial mechanism underlying impaired insulin release

Disruptions in the mitochondrial protein synthesis machinery give rise to metabolic disturbances that lay the foundation for type 2 diabetes. As physiological glucose levels rise, the energy-generating machinery of the mitochondria responds with increased activity, which stimulates insulin secretion. Many proteins responsible for mitochondrial metabolism are produced by ribosomes within this cellular organelle. Researchers led by Hyun Jin Kim and Minho Shong at Chungnam National University, Daejon, South Korea, have determined that mutations affecting a mitochondrial ribosomal protein called CRIF1 can lead to impaired insulin release. Mice with reduced CRIF1 were initially healthy, but as they aged, exhibited signs of impaired pancreatic function similar to those seen in patients with early-stage diabetes. This process was accelerated by consumption of a high-fat diet, and the researchers propose that this mechanism may be directly relevant to human disease.

## Introduction

Systemic glucose homeostasis requires adequate functional islet mass to provide appropriate glucose-stimulated insulin secretion (GSIS). Glucose-stimulated mitochondrial ATP production is required for the regulated exocytosis of insulin from β cells^1^. Mitochondrial ATP production is performed by the oxidative phosphorylation (OXPHOS) multiprotein complex^2^. The expression of mitochondrial DNA-encoded OXPHOS subunits is regulated predominantly at the posttranscriptional level^3^, which is performed by a specialized mitochondrial ribosome (mitoribosome) and associated factors^4^.

The mitoribosome is essential for cell viability, growth, differentiation, and function^5,6^, and loss or mutation of any of the mitoribosomal components can affect mitoribosomal RNA processing and can lead to mitochondrial disorders in humans^7^. In fact, the expression of the genes that encode mitoribosomal proteins (MRPs), mitoribosomal assembly factors, and mitochondrial translation factors is modified in numerous diseases^8,9^. Mutations of a single MRP frequently do not fully inactivate the mitoribosome but result in a lower capacity for oxidative phosphorylation. Although defects in any of the MRP genes could induce primary mitochondrial disease, only a small number of MRP mutations (MRPL9, MRPL27, and MRPL45) have been shown to be associated with diabetes^7^. This finding suggests that mutations in MRP genes may also result in tissue-specific phenotypes, giving rise to a spectrum of disorders, including diabetes. However, the influence of a single MRP on islet structure and systemic glucose tolerance has not previously been investigated. To clarify the role of the mitoribosome in β cells, it is necessary to determine how islets or β cells structurally and/or functionally adapt to the expression of MRP components.

CR6-interacting factor 1 (CRIF1), also known as MRPL59, is an MRP that forms part of the central protuberance of the large mitoribosomal subunit^10,11^. CRIF1 is essential for the synthesis and insertion of the OXPHOS complex into mammalian mitochondrial membranes^11^. Homozygous deficiency of the *Crif1* gene leads to both aberrant synthesis and defective insertion of mtDNA-encoded nascent OXPHOS polypeptides into the inner membrane^11^, and homozygous deficiency of *Crif1* in pancreatic β cells causes early islet failure in mice^12^. In view of the important role of the mitoribosome in the biogenesis of OXPHOS complexes in metabolic homeostasis, we aimed to determine whether MRPs in islets are associated with β-cell dysfunction and aberrant glucose homeostasis. To this end, we found that β-cell heterozygous *Crif1* deficiency was associated with an impairment of first-phase insulin secretion and consequent compensatory hyperplasia of islets. Furthermore, haploinsufficiency of *Crif1* in β cells resulted in earlier islet failure in mice fed a high-fat diet. Thus, this study provides conclusive genetic and functional evidence that a defect in an MRP is associated with glucose intolerance and islet pathology that is reminiscent of the early stage of human type 2 diabetes.

## Material and methods

### Analysis of differential gene expression using the GEO database

Bioinformatic analyses were performed using gene set enrichment analysis (GSEA) (http://www.broadinstitute.org/gsea) and R packages as described previously^13^. The publicly available transcriptomic datasets for human pancreatic islets were obtained from the NCBI Gene Expression Omnibus (GEO) site under accession numbers GSE25724, GSE76895, GSE76894, and GSE159984. The publicly available transcriptomic datasets for human pancreatic β cells were obtained from the European Nucleotide Archive under the accession number ERP017126^14^ (described in the Supplementary Information).

### Animals

Floxed Crif1 (Crif1^f/f^) mice were mated with Ins2-Cre transgenic mice to generate pancreatic β-cell-specific Crif1 heterozygous mice (*Crif1*^*beta+/−*^). Crif1^f/f^ mice were generated as described previously^15^. Ins2-Cre transgenic mice (C57BL/6-Tg(Ins2-cre)25Mgn/J) were purchased from the Jackson Laboratory (RRID:IMSR_JAX:003573). Ins2-Cre Tg mice express tryptophan hydroxylase and serotonin^16^. In all experiments, Cre-positive mice were used as a control. All mice were maintained in a controlled environment (12-h light/dark cycle; humidity, 50–60%; ambient temperature, 22 ± 2 °C) and fed a normal chow diet (NCD) or high-fat diet (HFD) (60% of calories from fat; Research Diets, D12492, New Brunswick, NJ, USA). All animal experiments were approved by the Committee on the Ethics of Animal Experiments of Chungnam National University Graduate School of Medicine (CNUH-017-A0048, Daejeon, Korea) and were performed according to the institutional guidelines for the care and use of laboratory animals.

### Islet isolation

The isolation of pancreatic islets was performed as previously described, with slight modifications^17^. Islets were isolated from mice by collagenase P (Roche Diagnostics, Mannheim, Germany) digestion and Ficoll (Biocoll separating solution, Biochrom, Berlin, Germany) gradient centrifugation (described in detail in the Supplementary Information).

### RNA isolation and quantitative real-time PCR (qPCR)

Total RNA was extracted from isolated islets, and qPCR was performed as described in the Supplementary Material. The PCR primers used are shown in Supplementary Table 1.

### Western blot analysis

Protein was extracted from isolated islets, and western blotting was performed as described in the Supplementary Material. The antibodies used are shown in Supplementary Table 2.

### Assessment of mitochondrial oxygen consumption

The oxygen consumption rate (OCR) of the islets was measured using a Seahorse XF-24 according to the manufacturer’s instructions (Seahorse Bioscience, North Billerica, MA, USA)^18^. The OCR was normalized to the mean baseline measurement in 2.8 mmol/l glucose and is expressed as a percentage change from baseline.

### Physiological and metabolic analyses

Perifusion analysis was performed in isolated islets. Islets from age-matched mice were isolated and perfused in sequence with Krebs–Ringer buffer (KRB) containing 2.8 mmol/l glucose for 10 min, followed by KRB containing 11.1 mmol/l glucose for 30 min, and finally KRB containing 40 mmol/l KCl for 5 min. The flow-through was collected every minute during the two perifusion steps of the assay, and the insulin concentrations of these solutions were measured.

The intraperitoneal glucose tolerance test (IPGTT) and the intraperitoneal insulin tolerance test (IPITT) were performed as described in the Supplementary Information.

### Transmission electron microscopy (TEM)

TEM was performed using *Crif1*^*beta+/−*^ and *Crif1*^*beta+/−*^ islets (described in detail in the Supplementary Information).

### Histological analysis

Formalin-fixed paraffin-embedded pancreatic slides were prepared, stained and analyzed (described in detail in the Supplementary Information).

### RNA-sequencing, data processing, and analysis

RNA sequencing was performed using *Crif1*^*beta+/−*^ and *Crif1*^*beta+/−*^ islets (described in detail in the Supplementary Information). The raw data for the RNA sequencing reported in this paper have been deposited to the NCBI GEO repository (GSE151708).

### Statistical analysis

Statistical analyses were performed using SPSS Version 21 (IBM, Inc., Armonk, NY, USA). We used an unpaired, two-tailed Student’s *t* test for comparisons between two groups and one-way ANOVA followed by Tukey’s HSD test for multiple comparisons between three or more groups. Data are expressed as the means ± SEMs. *p* < 0.05 was considered statistically significant.

## Results

### MRPs in human pancreatic islets are associated with the development of type 2 diabetes

**Fig. 1:**
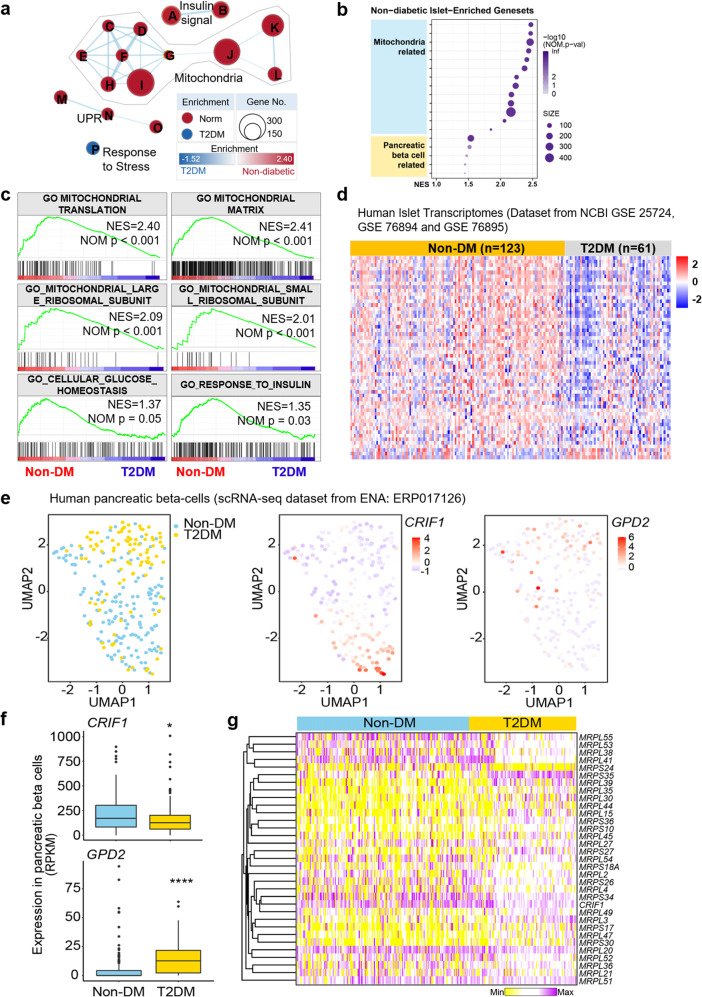
In silico analysis reveals an association between type 2 diabetes and the mitoribosomal protein CRIF1 in human pancreatic islets. **a–d** Results of gene set enrichment analysis (GSEA) of GSE25724. **a** Visualization of the enrichment map. Red, gene set enriched in nondiabetic (non-DM) individuals; blue, gene set enriched in type 2 diabetes mellitus (T2DM) subjects. The thickness of each line represents the strength of the correlation between nodes, and the size of each circular node represents the size of the gene set (A: Gene ontology (GO) CELLULAR RESPONSE TO INSULIN STIMULUS, B: GO RESPONSE TO INSULIN, C: GO MITOCHONDRIAL TRANSLATIONAL TERMINATION, D: GO MITOCHONDRIAL TRANSLATION, E: REACTOME MITOCHONDRIAL TRANSLATION, F: GO MITOCHONDRIAL LARGE RIBOSOMAL SUBUNIT, G: GO MITOCHONDRIAL SMALL RIBOSOMAL SUBUNIT, H: GO MITOCHONDRIAL GENE EXPRESSION, I: GO MITOCHONDRIAL MATRIX, J: MOOTHA MITOCHONDRIA, K: MITOCHONDRION, L: MITOCHONDRIAL PART, M: GO ENDOPLASMIC RETICULUM UNFOLDED PROTEIN RESPONSE, N: REACTOME UNFOLDED PROTEIN RESPONSE (UPR), O: HALLMARK UPR, P: GO MULTICELLULAR ORGANISMAL RESPONSE TO STRESS). **b** Bubble plot showing the normalized enrichment score for each gene set related to mitochondrial and pancreatic β-cell functions. **c** Enrichment plots for selected gene sets. **d** Heatmap showing the mitoribosomal protein (MRP) gene expression patterns for the three GSEA sets. **e** UMAP plots showing the origin (i.e., non-DM and T2DM, left) and displaying *CRIF1* (center) and *GPD2* (right) expression in each human pancreatic β cell. **f** Boxplots showing the median (2nd and 3rd quartiles) expression of *CRIF1* and *GPD2*. **g** Heatmap showing MRP gene expression in individual human pancreatic β cells.

To investigate the association between type 2 diabetes and MRPs, including *CRIF1*, in human pancreatic islets, we analyzed the dataset on the NCBI GEO website (GSE25724), which contains the transcriptomes of type 2 diabetic and nondiabetic organ donors^19^. An unbiased GSEA revealed that all the identified mitochondrial gene sets were enriched in human nondiabetic islets, as were the gene sets related to pancreatic β-cell function (Fig. 1a, b and Supplementary Fig. 1a). For instance, the GO MITOCHONDRIAL TRANSLATION gene set and the GO MITOCHONDRIAL MATRIX gene set were enriched in nondiabetic human islets, as were gene sets related to pancreatic β-cell function (Fig. 1c). In addition, the majority of MRPs, which are included in the GO_MITOCHONDRIAL_LARGE_RIBOSOMAL_SUBUNIT and GO_MITOCHONDRIAL_SMALL_RIBOSOMAL_SUBUNIT gene sets, were enriched in nondiabetic islets (Fig. 1c). We found that the expression of MRP genes in type 2 diabetic human islets was significantly different in GSE25724.

Next, to avoid possible misinterpretation because of confounding factors in the single dataset analysis, we collected transcriptomic datasets that included gene expression in human pancreatic β cells from 184 individuals (GSE25724^19^, GSE76894, and GSE76895^20^) and conducted a meta-analysis of the transcriptomic data. All the collected data were uniformly preprocessed and converted to *Z* scores to avoid batch effects^21,22^. The resulting heatmap (Fig. 1d) indicated that the expression of these MRPs clearly differentiates islets from type 2 diabetic and nondiabetic patients. In particular, the Crif1 gene showed a clear reduction in the T2DM patients and is visualized in the boxplot (Supplementary Fig. 1b). To avoid any potential confounding factor from microarray-based transcriptome analysis, we collected and analyzed the high-throughput transcriptome-sequencing dataset of islets donated from type 2 diabetic and nondiabetic donors generated by Marselli et al., which is available at NCBI GEO (GSE159984)^23^ (Supplementary Fig. 2a). The raw read count data were normalized to transcripts per million (TPM), followed by logarithmic transformation, as summarized in Supplementary Fig. 2a. Then, we conducted enrichment and pathway analysis to identify gene sets enriched in type 2 diabetic and nondiabetic donors by GSEA. The results of the analyses, including the enrichment of mitochondria and their related components, were highly consistent with the GSEA results discussed above (Supplementary Fig. 2b–d). In addition, global MRP expression was different in type 2 diabetic and nondiabetic donors (Supplementary Fig. 2e).

The results discussed above consistently suggest that the dysregulation of MRPs in the pancreas is associated with type 2 diabetes. To overcome the limitation of the global transcriptomic analysis, which could be a confounding factor in understanding the role of MRPs in pancreatic β cells rather than pancreatic islets, we further analyzed the publicly available scRNA-seq data^14^ (Accession Number: ERP017126). The UMAP algorithm clustered pancreatic β cells into two subpopulations that matched the disease status (Fig. 1e, left). The expression of CRIF1 was relatively higher in the non-DM group (Fig. 1e, center), whereas the expression of the glycerol-3-phosphate dehydrogenase 2 (*GPD2*) gene was elevated in the T2DM group (Fig. 1e, right), as indicated in the original publication of the scRNA-seq study (see the supplementary data of ref. ^14^). The gene expression levels of both *CRIF1* and *GPD2* were visualized in boxplots and showed a clear reduction and induction in T2DM, respectively (Fig. 1f). Furthermore, not only *CRIF1* but also most MRP expression was altered in human diabetic pancreatic β cells (Fig. 1g).

### Crif1 haploinsufficiency in β cells is associated with lower glucose-stimulated respiratory efficiency

**Fig. 2:**
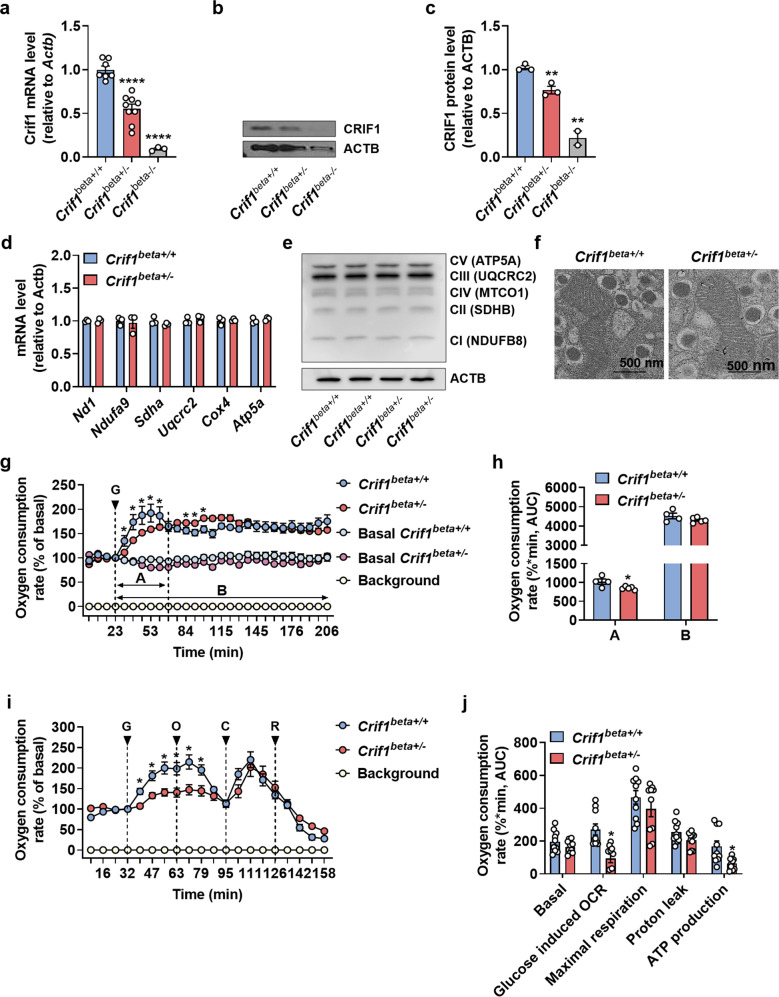
*Crif1* haploinsufficiency in β cells is associated with impaired glucose-stimulated respiration. **a**–**c** CRIF1 expression in islets from 14-week-old mice. **a** Relative mRNA expression of *Crif1*, measured using qRT–PCR (*n* **=** 3−9) and normalized to *Actb* expression. **b** Representative western blot of CRIF1. **c** ImageJ quantification of CRIF1 protein expression in *Crif1*^*beta+/+*^*, Crif1*^*beta+/−*^, and *Crif1*^*beta−/−*^ mice (*n* **=** 2−3). **d** and **e** Oxidative phosphorylation (OXPHOS) complex expression in islets from 14-week-old *Crif1*^*beta+/−*^ isolated islets. **d** Relative mRNA expression of OXPHOS genes in islets from *Crif1*^*beta+/+*^ and *Crif1*^*beta+/−*^ mice (*n* **=** 3), normalized to *Actb* (*Nd1*, *Ndufa9*: complex 1, *Sdha*: complex 2, *Uqcrc2*: complex 3, *Cox4*: complex 4, and *Atp5A*: complex 5). **e** Protein expression of OXPHOS components in islets from *Crif1*^*beta+/+*^ and *Crif1*^*beta+/−*^ mice normalized to ACTB. **f** Representative transmission electron micrographs (TEMs) of β-cell mitochondria from 14-week-old *Crif1*^*beta+/+*^ and *Crif1*^*beta+/−*^ mice. Scale bars: 500 nm. **g** Glucose-induced oxygen consumption rate (OCR) in islet mitochondria from 14-week-old *Crif1*^*beta+/+*^ and *Crif1*^*beta+/−*^ mice (*n* **=** 4−6). The treatment was 20 mmol/l glucose (G). **h** Area under the curve (AUC) for each phase of the glucose-induced OCR. **i** OCR in islet mitochondria from 14-week-old *Crif1*^*beta+/+*^ and *Crif1*^*beta+/−*^ mice (*n* **=** 6−10). The treatments were 20 mmol/l glucose (G), 2 μmol/l oligomycin (O) (an inhibitor of ATP synthase), 5 μmol/l carbonyl cyanide *m*-chlorophenyl hydrazone (CCCP) (C) (an uncoupler), and 2 mmol/l rotenone (R) (an inhibitor of complex I). **j** AUC for each phase of the OCR. Data are the mean ± SEM. *p **<** 0.05, **p **<** 0.005, ***p **<** 0.005, and ****p **<** 0.0001 vs. *Crif1*^*beta+/+*^.

To investigate the associations of MRP expression with β-cell function and glucose tolerance, we generated a pancreatic β-cell-specific *Crif1* knockout mouse by crossing floxed *Crif1* mice with mice carrying *Ins2*-cre recombinase. qPCR analysis of isolated islets showed that *Crif1* mRNA expression in *Ins2-Crif1* heterozygous (*Crif1*^*beta+/−*^) mice was half that of *Ins2*-cre control (*Crif1*^*beta+/+*^) mice at 14 weeks old (Fig. 2a). Additionally, the expression of CRIF1 protein in islets of *Crif1*^*beta+/−*^ mice was ~30% lower than that of *Crif1*^*beta+/+*^ mice as assessed by western blotting at 14 weeks old (Fig. 2b, c). However, the expression of selected OXPHOS polypeptides that are encoded by both mitochondrial and genomic genes was similar in the two genotypes at 14 weeks old (Fig. 2d, e). Moreover, electron microscopy demonstrated that the number, size, and structure of the β-cell mitochondria were similar in *Crif1*^*beta+/+*^ and *Crif1*^*beta+/−*^ mice at 14 weeks old (Fig. 2f, Supplementary Fig. 3a, b).

To evaluate the effect of *Crif1* haploinsufficiency on cellular respiration, we measured the OCR of islets isolated from both *Crif1*^*beta+/−*^ and *Crif1*^*beta+/+*^ mice at 14 weeks old. We assessed glucose-stimulated mitochondrial function by treating islets with 20 mmol/l glucose and performing serial measurements of the OCR for the following 3 h. Islets from both *Crif1*^*beta+/+*^ and *Crif1*^*beta+/−*^ mice showed similar OCRs in the presence of 3 mmol/l (basal) glucose. However, islets isolated from *Crif1*^*beta+/−*^ mice did not show a high glucose-induced peak (represented as phase A) in glucose-stimulated (20 mmol/l) oxygen consumption, unlike those from control *Crif1*^*beta+/+*^ mice (Fig. 2g, h). This result indicates that islets of *Crif1*^*beta+/−*^ mice have a lower glycolytic or oxidative capacity and a lower ability to respond appropriately to a high glucose concentration. To determine whether islets from *Crif1*^*beta+/−*^ mice have lower oxidative capacity, we measured the OCR in the presence of oligomycin (an ATP synthase inhibitor), carbonyl cyanide *m*-chlorophenyl hydrazone (CCCP; a mitochondrial respiration uncoupler), or rotenone (an OXPHOS complex I inhibitor) in buffer containing basal or high glucose concentrations (3 and 20 mmol/l glucose, respectively). *Crif1*^*beta+/−*^ islets showed normal basal oxygen consumption but did not show a high glucose-induced peak in oxygen consumption in the presence of a high glucose concentration, unlike *Crif1*^*beta+/+*^ islets. The inhibition of ATP synthase by oligomycin prevented the stimulation of oxygen consumption by 20 mmol/l glucose. Interestingly, the oxygen consumption responses to CCCP (5 µmol/l) and rotenone (2 mmol/l) did not differ between control and *Crif1*^*beta+/−*^ islets (Fig. 2i). Taken together, although β cells in islets from *Crif1*^*beta+/−*^ mice showed no apparent mitochondrial structural abnormalities, they demonstrated an impairment in glucose-stimulated mitochondrial ATP production (Fig. 2i, j).

### *Crif1*^*beta+/−*^ mice show an age-dependent decline in glucose tolerance

**Fig. 3:**
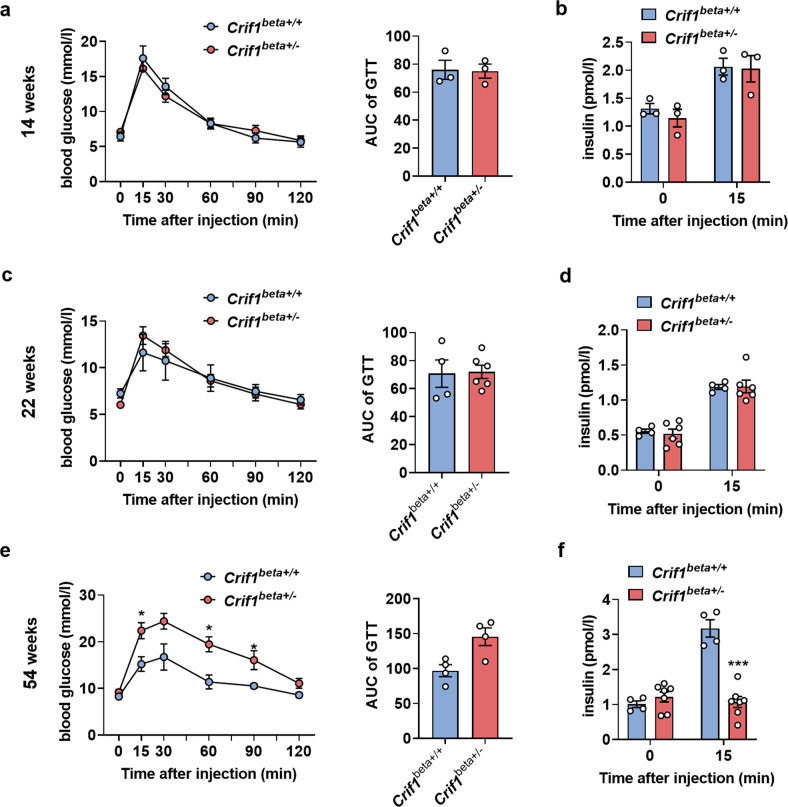
*Crif1*^*beta +/−*^ mice show an age-related loss of glucose tolerance. **a**, **c**, and **e** Blood glucose concentrations during intraperitoneal glucose tolerance testing (IPGTT) (6-h fast; 1 g/kg glucose dose) performed in *Crif1*^*beta+/+*^ and *Crif1*^*beta+/−*^ mice (*n* = 3−7). The areas under the curves (AUCs) during the IPGTT are shown for **a** 14-week-old, **c** 22-week-old, and **e** 54-week-old mice. **b**, **d**, **f** Glucose-stimulated insulin secretion (GSIS) was assessed during the IPGTT in *Crif1*^*beta+/+*^ and *Crif1*^*beta+/−*^ mice (*n* = 3−7) at **b** 14 weeks of age, **d** 22 weeks of age, and **f** 54 weeks of age. Data are the mean ± SEM. *p < 0.05 vs. *Crif1*^*beta+/+*^.

To determine whether *Crif1* haploinsufficiency is associated with β-cell dysfunction and aberrant glucose homeostasis during aging, we performed glucose tolerance tests in *Crif1*^*beta+/−*^ and control mice at 14, 22, and 54 weeks of age. *Crif1*^*beta+/−*^ and *Crif1*^*beta+/+*^ mice showed normal glucose tolerance (Fig. 3a, c) and similar serum insulin concentrations upon fasting or 15 min after glucose administration (Fig. 3b, d) at both 14 and 22 weeks. Furthermore, the mean body mass of the two groups of mice was similar between 14 and 22 weeks of age (Supplementary Fig. 4a); the insulin sensitivity of *Crif1*^*beta+/−*^ mice was normal at 22 weeks of age (Supplementary Fig. 4b). However, 54-week-old *Crif1*^*beta+/−*^ mice were glucose intolerant (Fig. 3e), showed a poor insulin secretory response to glucose (Fig. 3f), and had a significantly higher body mass than control mice (Supplementary Fig. 4a). The appearance of glucose intolerance in *Crif1*^*beta+/−*^ mice at 54 weeks of age suggests that haploinsufficiency of a mitoribosomal subunit gene in islets promotes aging-associated islet dysfunction.

### Islets from *Crif1*^*beta+/−*^ mice show a loss of first-phase insulin secretion before the onset of glucose intolerance

**Fig. 4:**
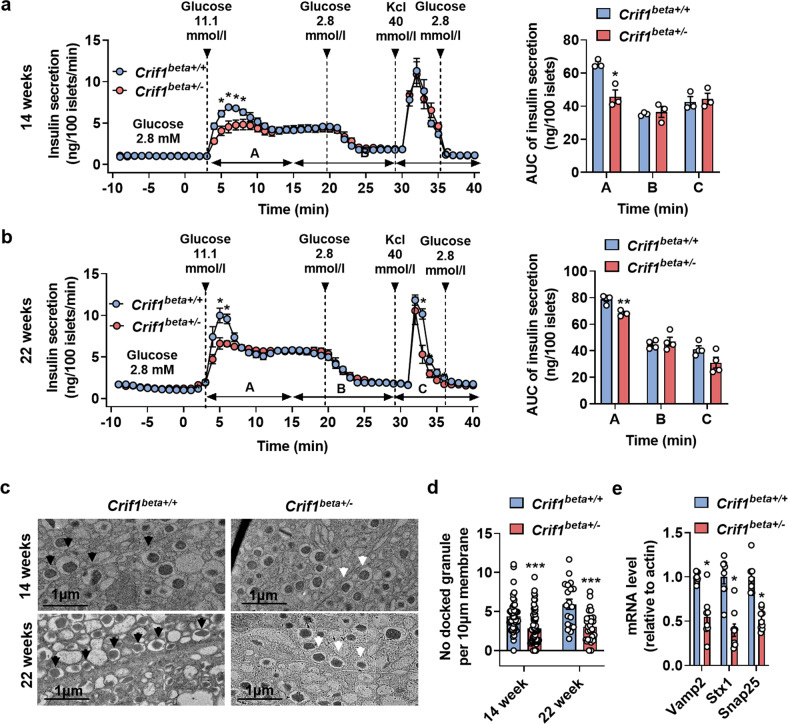
Islets from *Crif1*^*beta+/−*^ mice do not show first-phase insulin secretion. **a** and **b** Isolated islets from *Crif1*^*beta+/+*^ and *Crif1*^*beta+/−*^ were perifused with 2.8 or 11.1 mmol/l glucose, with or without 40 mmol/l KCl. Areas under the curves (AUCs) for A = 3–15 min, B = 14–28 min, and C = 28–40 min (*n* = 3) in **a** 14-week-old and **b** 22-week-old mice. **c** Representative transmission electron micrographs (TEMs) of insulin granules in β cells from 14- and 22-week-old *Crif1*^*beta+/+*^ and *Crif1*^*beta+/−*^ mice. Docked granules highlighted using arrows; scale bar: 1 μm. **d** Number of docked insulin granules, quantified as the number of granules found within 100 nm of the plasma membrane (per 10 μm^2^ area) in 14- and 22-week-old *Crif1*^*beta+/+*^ and *Crif1*^*beta+/−*^ β cells. **e** Relative mRNA expression of docking-related genes in isolated islets from 14-week-old *Crif1*^*beta+/+*^ and *Crif1*^*beta+/−*^ mice normalized to *Actb* expression. Data are the mean ± SEM. *p < 0.05, **p < 0.005, and ***p < 0.005 vs. *Crif1*^*beta+/+*^.

An insulin perifusion assay was performed to characterize insulin release by islets isolated from 14- and 22-week-old mice that were not glucose intolerant. This assay showed that first-phase insulin secretion (within 10 min) was >20% lower in islets from *Crif1*^*beta+/−*^ mice than in control islets. However, the insulin secretion by the islets did not differ after 10 min of the perifusion assay, and there was no difference in the maximal insulin release by the islets after KCl-induced depolarization between the genotypes. In addition, the area under the curves (AUCs) of insulin release during the perifusion assay confirmed that it differed significantly between the groups from 3 to 15 min following glucose stimulation but not after 15 min (Fig. 4a, b). Moreover, the islet insulin content did not differ between *Crif1*^*beta+/−*^ and *Crif1*^*beta+/+*^ islets (Supplementary Fig. 5a, b). Measurement of [Ca^2+^]_i_ by fluorescence imaging of Fura-2-loaded islets revealed that the [Ca^2+^]_i_ responses to glucose and KCl stimulation did not differ between *Crif1*^*beta+/−*^ and *Crif1*^*beta+/+*^ islets (Supplementary Fig. 5c). We next examined the insulin granules of isolated islets using TEM and found that there were fewer readily releasable pools (RRPs) of granules near the membrane in *Crif1*^*beta+/−*^ islets (approximately three per 10 μm of plasma membrane) than in *Crif1*^*beta+/+*^ islets (approximately five per 10 μm of plasma membrane) from 14- and 22-week-old mice (Fig. 4c, d). The mRNA expression of *Vamp2*, *Stx1*, and *Snap25*, which are genes that encode proteins involved in the docking of granules, was lower in *Crif1*^*beta+/−*^ islets from 14-week-old mice (Fig. 4e). These results suggest that *Crif1* haploinsufficiency interferes with the docking and exocytosis of granules, resulting in a defect in first-phase insulin secretion that worsens with age.

### *Crif1*^*beta+/−*^ mice develop age-dependent islet hyperplasia in the period of normal glucose tolerance

**Fig. 5:**
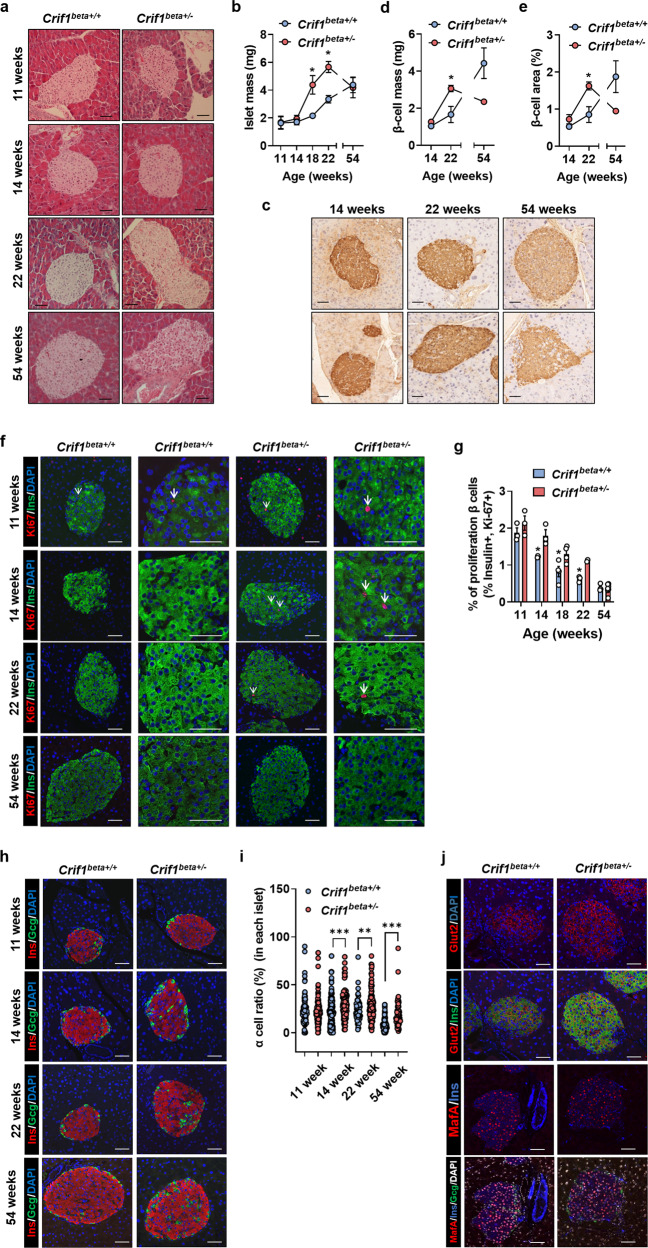
*Crif1*^*beta+/−*^ mice show abnormal islet morphology. **a** and **b** Islet area in pancreatic sections from 11-, 14-, 22-, and 54-week-old *Crif1*^*beta+/+*^ and *Crif1*^*beta+/−*^ mice. **a** Representative images of hematoxylin and eosin (H&E)-stained islets from 11-, 14-, 22-, and 54-week-old *Crif1*^*beta+/+*^ and *Crif1*^*beta+/−*^ mice. **b** Islet mass quantification (*n* = 4−6). **c–e** β-Cell mass of pancreatic sections from 14-, 22-, and 54-week-old *Crif1*^*beta+/+*^ and *Crif1*^*beta+/−*^ mice. **c** Representative immunohistochemistry images showing β-cell mass. **d** and **e** β-Cell mass quantification (*n* = 3). **f** and **g** β-Cell proliferation rate on pancreatic sections from 11-, 14-, 22-, and 54-week-old *Crif1*^*beta+/+*^ and *Crif1*^*beta+/−*^ mice. **f** Representative immunofluorescent images showing β-cell proliferation (proliferation marker Ki67: red, insulin (Ins): green, and DAPI: blue). White arrows indicate Ki67-positive cells. **g** β-Cell proliferation rate, expressed as a percentage of the β cells that were proliferating (Ki-67-positive nuclei/β-cell count × 100%, analyzed per mouse). **h** and **i** α/β-Cell numerical ratio in pancreatic sections from 11-, 14-, 22-, and 54-week-old *Crif1*^*beta+/+*^ and *Crif1*^*beta+/−*^ mice. **h** Representative immunofluorescent islet images (β-cell marker Ins: red, α-cell marker glucagon (Gcg): green, and DAPI: blue). **i** α-Cell ratio (α-cell number/whole islet cell number*100%). **j** Representative immunofluorescent images of pancreatic sections showing β cells in islets from *Crif1*^*beta+/+*^ and *Crif1*^*beta+/−*^ mice at 22 weeks of age. Upper: mature β-cell marker glucose transporter 2 (Glut2): red, β-cell marker Ins: green, and DAPI: blue. Lower: Glut2: red, Ins: blue, Gcg: green, and DAPI: white. Data are the mean ± SEM. *p < 0.05, **p < 0.005, and ***p < 0.0005. Scale bar: 50 μm.

To characterize any histological changes, pancreatic sections were prepared at 11, 14, 22, and 54 weeks of age and stained using H&E (Fig. 5a). The islet mass of *Crif1*^*beta+/−*^ mice increased until 22 weeks of age, and while the area did not differ significantly from the control group up to 14 weeks of age, it was 4-fold larger at 18 and 22 weeks of age (Fig. 5b). The β-cell mass (mg) and β-cell area (%) of *Crif1*^*beta+/−*^ mice did not differ significantly from those of the control group at 14 weeks of age but were 2-fold greater at 22 weeks of age (Fig. 5c–e).

Interestingly, at 54 weeks of age, the islet mass of *Crif1*^*beta+/*−^ mice decreased to levels similar to those of *Crif1*^*beta+/+*^ mice (Fig. 5b), and β-cell mass was lower than that of *Crif1*^*beta+/+*^ mice (Fig. 5d). The number of islets and the weight of the pancreas were similar in the two groups at 11, 14, 22, and 54 weeks of age (Supplementary Fig. 6a, b). Analysis of the islet size distribution showed that the proportion of large islets in *Crif1*^*beta+/+*^ mice increased until 22 weeks of age (Supplementary Fig. 6c), but this difference was not present at 54 weeks (Supplementary Fig. 6d–f). These findings indicate that *Crif1* haploinsufficiency increases the number of larger islets, islet mass, and β-cell mass, but this hyperplasic response is not maintained in aged mice.

To assess the islet cell proliferation rate, dual immunofluorescence (IF) staining for insulin and the proliferation marker Ki-67 was performed in pancreatic sections from 11-, 14-, 18-, 22-, and 54-week-old mice (Fig. 5f). The rate of β-cell proliferation gradually decreased with age in both *Crif1*^*beta+/+*^ and *Crif1*^*beta+/−*^ mice (Fig. 5g). The rate of β-cell proliferation in the *Crif1*^*beta+/−*^ mice was higher than that in the *Crif1*^*beta+/+*^ mice at 11, 14, 18, and 22 weeks of age but similar at 54 weeks of age (Fig. 5g). Taken together, these data show that *Crif1* haploinsufficiency causes islet hyperplasia, but this does not persist into advanced age.

### Islets of *Crif1*^*beta+/−*^ mice have a high α-cell numerical ratio

We next assessed islet composition by staining pancreatic sections from 11-, 14-, 22-, and 54-week-old mice for insulin and glucagon (Fig. 5h). The islets of 11-week-old *Crif1*^*beta+/−*^ mice had a normal distribution of glucagon-positive α cells and insulin-positive β cells in the periphery and core of the pancreas, respectively, with no difference in the numerical ratio of α cells between the groups (Fig. 5h, i). Interestingly, the islets of *Crif1*^*beta+/−*^ mice had a higher α-cell ratio and a higher central distribution of α cells at 14 and 22 weeks of age than control mice (Fig. 5h, i). However, the basal and glucose-stimulated plasma glucagon concentrations in *Crif1*^*beta+/−*^ mice were similar to those in *Crif1*^*beta+/+*^ mice (Supplementary Fig. 7). Additionally, the expression of two maturation markers of β cells, namely, V-maf musculoaponeurotic fibrosarcoma oncogene homolog A (MAFA) and glucose transporter 2 (GLUT2), was similar in both *Crif1*^*beta+/−*^ and *Crif1*^*beta+/+*^ mice (Fig. 5j). Taken together, *Crif1* haploinsufficiency in β cells results in an increase in the relative number of α cells but does not affect glucose tolerance or glucagon secretion in unchallenged animals.

### Islets of *Crif1*^*beta+/−*^ mice show high expression of Reg family genes

**Fig. 6:**
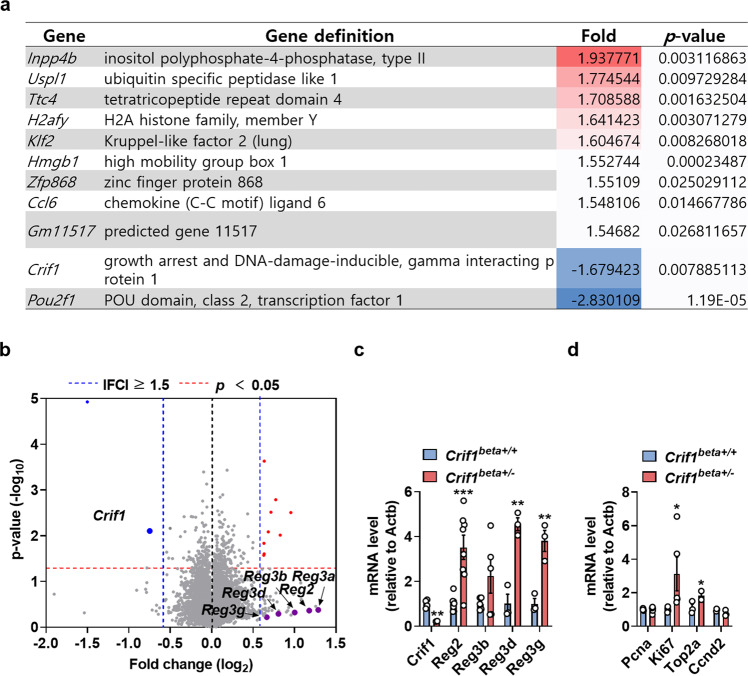
The expression of *Reg* family genes is high in islets from *Crif1*^*beta+/−*^ mice. **a** and **b** RNA-sequencing data for isolated islets from *Crif1*^*beta+/+*^ and *Crif1*^*beta+/−*^ mice (*n* = 3). **a** List of differentially expressed transcripts (raw data, ≥1.5-fold difference; *p* < 0.05). **b** Volcano plot showing the significantly upregulated (red) and downregulated (blue) genes. **c** Relative mRNA expression of *Reg* family genes in isolated islets from 14-week-old *Crif1*^*beta+/+*^ and *Crif1*^*beta+/−*^ mice normalized to *Actb* expression. **d** Relative mRNA expression of the proliferation markers *Pcna*, *Ki67*, *Top2a*, and *Ccnd2* in isolated islets from 14-week-old *Crif1*^*beta+/+*^ and *Crif1*^*beta+/−*^ mice normalized to *Actb* expression. Data are the mean ± SEM. *p < 0.05.

To investigate the molecular mechanism of the high islet mass in *Crif1*-haploinsufficient mice, we compared the global gene expression patterns of *Crif1*^*beta+/−*^ and *Crif1*^*beta+/+*^ mouse islets at 14 weeks of age. Seventy-two out of 7966 genes were differentially expressed (fold difference > 1.5) in *Crif1*^*beta+/−*^ islets. The expression of 56 genes was 1.5-fold higher in *Crif1*^*beta+/−*^ islets, while 16 genes were 1.5-fold lower. Of these, only 11 genes were significantly differentially expressed (fold change > 1.5, raw data; *p* < 0.05) (Fig. 6a). The *Reg* gene family (*Reg2, Reg3a, Reg3b, Reg3d*, and *Reg3g*) exhibited robust differences in expression and is shown in the volcano plot (Fig. 6b). *Reg2, Reg3b, Reg3d*, and *Reg3g* mRNA expression was approximately 2–4-fold higher in islets from *Crif1*^*beta+/−*^ mice than in *Crif1*^*beta+/+*^ mice (Fig. 6c). The *Reg* family is known to be involved in islet regeneration. To confirm an increase in islet proliferation, we performed qPCR analysis of the mRNA expression of proliferation markers (*Pcna, Ki-67, Top2a*, and *Ccnd2*) and found that the mRNA expression of *Ki-67* and *Top2a* was higher in *Crif1*^*beta+/−*^ islets (Fig. 6d). Thus, genes related to cell proliferation (*Reg* family, *Ki-67*, and *Pcna*) were expressed at significantly higher levels, which may explain the greater islet mass in *Crif1*^*beta+/−*^ mice.

### *Crif1*^*beta+/−*^ mice show earlier β-cell failure when fed a high-fat diet

**Fig. 7:**
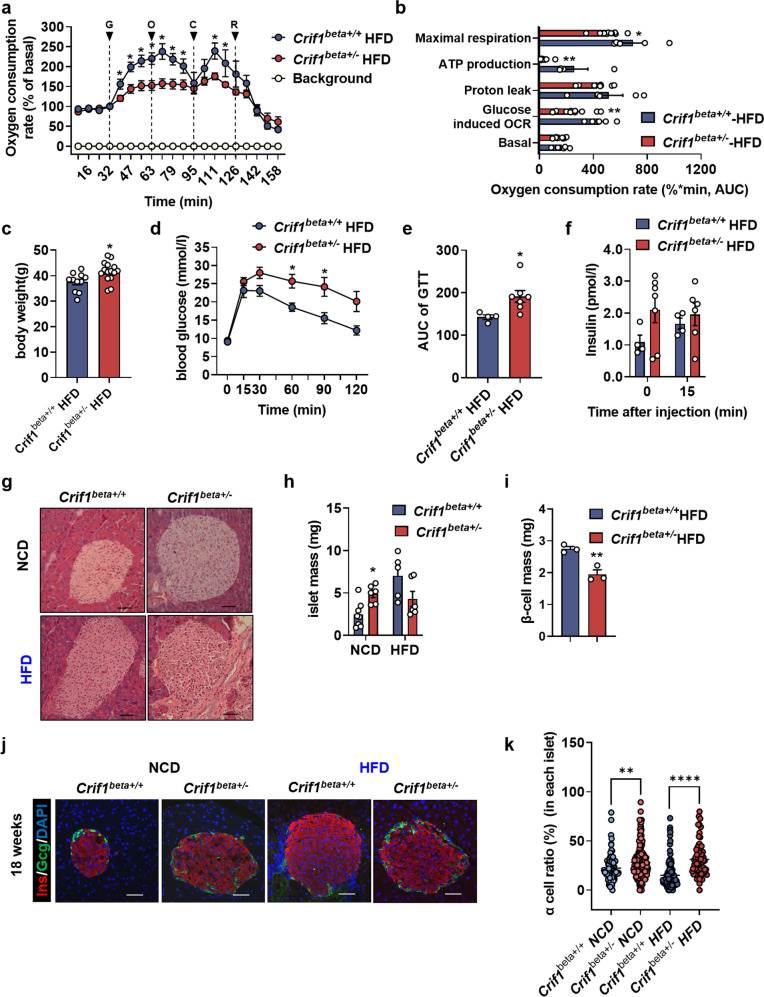
*Crif1* haploinsufficient mice are susceptible to metabolic stress. **a** The oxygen consumption rate (OCR), measured in isolated islets from high-fat diet (HFD)-fed *Crif1*^*beta+/+*^ and *Crif1*^*beta+/−*^ mice (*n* = 5−10). The treatments were 20 mmol/l glucose (G), 2 μmol/l oligomycin (O) (an inhibitor of ATP synthase), 5 μmol/l carbonyl cyanide *m*-chlorophenyl hydrazone (CCCP) (C) (an uncoupler), and 2 mmol/l rotenone (R) (an inhibitor of complex I). **b** The area under the curve (AUC) for each phase of the oxygen consumption measurement. **c** Twelve weeks of HFD consumption from 6 weeks of age increased the body mass of *Crif1*^*beta+/+*^ and *Crif1*^*beta+/−*^ mice (*n* = 4−7). **d** Blood glucose concentrations during intraperitoneal glucose tolerance testing (IPGTT) (6-h fast; 1 g/kg glucose dose) in 1-year-old *Crif1*^*beta+/+*^ and *Crif1*^*beta+/−*^ mice. **e** AUCs for the IPGTT. **f** Glucose-stimulated insulin secretion (GSIS) during the IPGTT. **g** Islet area, shown in representative images of hematoxylin and eosin-stained pancreatic sections. **h** Islet area quantification (*n* = 4−6). **i** β-cell mass quantification (*n* = 3). **j** Representative sections showing the islets of *Crif1*^*beta+/+*^ and *Crif1*^*beta+/−*^ mice at 18 weeks of age that had been fed a HFD for 12 weeks. Immunofluorescent staining: Ins, red; Gcg, green; and DAPI, blue. **k** α-Cell ratio (α-cell number/whole islet cell number*100), calculated for islets from *Crif1*^*beta+/+*^ and *Crif1*^*beta+/−*^ mice. Data are the mean ± SEM. *p < 0.05 and *p < 0.005 vs. *Crif1*^*beta+/+*^. Scale bar: 50 μm.

To examine the compensation for metabolic stress, we next analyzed mitochondrial respiration and the metabolic phenotype of 18-week-old *Crif1*^*beta+/−*^ and *Crif1*^*beta+/+*^ mice that had been fed a HFD for the preceding 12 weeks. Islets from *Crif1*^*beta+/−*^ mice fed a HFD showed lower glucose-induced OCR and responses to oligomycin and CCCP than those of the control group (Fig. 7a). In addition, ATP production (approximately 90%) and maximal respiratory capacity (approximately 30%) were significantly lower in *Crif1*^*beta+/−*^ islets (Fig. 7b). These data imply that *Crif1* haploinsufficiency in β cells impairs local mitochondrial respiration in mice fed a HFD.

To investigate the metabolic response of *Crif1*^*beta+/−*^ mice to HFD feeding, their body mass gain, glucose tolerance, and pancreatic histology were evaluated. The body mass gains of *Crif1*^*beta+/−*^ and *Crif1*^*beta+/+*^ mice on a HFD for 10 weeks were similar (Supplementary Fig. 8a). However, *Crif1*^*beta+/−*^ mice weighed significantly more than *Crif1*^*beta+/+*^ mice after 12 weeks of HFD consumption (Fig. 7c and Supplementary Fig. 8a). The IPGTT after 12 weeks of HFD feeding revealed a significant impairment in glucose disposal in *Crif1*^*beta+/−*^ mice relative to *Crif1*^*beta+/+*^ mice (Fig. 7d, e). Furthermore, the plasma insulin concentration of the former was higher after 6 h of fasting (time 0) but failed to show a further increase at 15 min after the glucose load in *Crif1*^*beta+/−*^ mice (Fig. 7f). The random blood glucose concentrations of the two groups were comparable (Supplementary Fig. 8b), but *Crif1*^*beta+/−*^ mice were less insulin-sensitive than *Crif1*^*beta+/+*^ mice because they had higher blood glucose concentrations at 30 and 60 min after insulin injection at the end of the 12-week period of HFD consumption (Supplementary Fig. 8c).

The effects of HFD feeding on pancreatic histology were assessed using H&E-stained sections (Fig. 7g). HFD-fed *Crif1*^*beta+/+*^ mice had a more than 2-fold larger pancreatic islet mass than normal chow diet (NCD)-fed *Crif1*^*beta+/+*^ mice to compensate for metabolic stress, but there was no difference in the pancreatic islet mass between HFD-fed and NCD-fed *Crif1*^*beta+/−*^ mice (Fig. 7g, h). In addition, β-cell mass was significantly lower in HFD-fed *Crif1*^*beta+/-*^ mice than in HFD-fed *Crif1*^*beta+/+*^ mice (Fig. 7i). These results indicate that *Crif1*^*beta+/−*^ mice fail to compensate for metabolic stress by means of islet hyperplasia. Analysis of the islet size distribution showed that the proportion of large islets was higher in NCD-fed *Crif1*^*beta+/−*^ mice but lower in HFD-fed *Crif1*^*beta+/−*^ mice (Supplementary Fig. 8d). Normal islets were circular and were found throughout the pancreatic sections, but HFD-fed *Crif1*^*beta+/−*^ mice were less circular and showed an increased presence of disorganized islets (Fig. 7g, j). Furthermore, *Crif1*^*beta+/−*^ mice showed higher centralized α cells and a higher α-cell ratio than HFD-fed control mice (Fig. 7j, k). In summary, *Crif1* haploinsufficiency in β cells renders mice vulnerable to metabolic stress and is associated with poor islet architecture and glucose intolerance.

## Discussion

We have shown that deficiency of a single MRP in pancreatic islets causes age- and HFD-associated islet failure, which is reminiscent of the islet phenotype of patients in the early stages of type 2 diabetes. Mice with haploinsufficiency of the *Crif1* gene display normal glucose tolerance in early life, with a marked compensatory increase in islet mass. Furthermore, there were marked differences in MRP gene expression in human islets and β cells from nondiabetic and diabetic individuals. Therefore, mitochondrial matrix proteostasis, which is mediated via the mitoribosome, may significantly influence the risk of developing type 2 diabetes. CRIF1 is a novel mitochondrial component that is located in the central protuberance region of the large subunit of the mitoribosome. We analyzed a single-cell RNA-seq dataset and found that the expression of *CRIF1* was reduced in human β cells, which is evidence that *CRIF1* could play an important pathophysiologic role in human diabetes. Furthermore, we provide evidence that deficiency of the *Crif1* gene is associated with islet failure, causing systemic glucose intolerance, in mice.

The present mouse model displays different features from those previously reported for mitochondrial diabetes, which is characterized by β-cell-specific mitochondrial dysfunction. Mice carrying such β-cell-specific genetic defects showed early-onset, severe diabetic phenotypes, such as poor GSIS, glucose intolerance, and low β-cell mass, followed by a marked reduction in mitochondrial function^24–26^. In particular, the *Crif1*^*beta−/*−^ model, which we reported previously, exhibited impaired glucose tolerance with defective insulin secretion as early as 4 weeks of age and decreased mitochondrial function, islet area, and insulin contents at 11 weeks of age^12^. These results suggest that homozygotic *Crif1*^*beta−/*−^ mice have the typical features of mice with mitochondrial diabetes. In contrast, the heterozygotic *Crif1*^*beta+/−*^ mice showed lower glucose-stimulated mitochondrial ATP production but normal overall GSIS. Furthermore, they had a 1.5-fold higher β-cell proliferation rate and an >2-fold larger islet mass at 22 weeks of age. Islet failure occurred in *Crif1*^*beta+/−*^ mice when they became older and were subjected to the metabolic insult of HFD feeding^27–29^. Thus, the islets of *Crif1*^*beta+/−*^ mice demonstrate features that are typical of the early stages of type 2 diabetes, including loss of first-phase insulin secretion, islet hyperplasia, and altered cellular composition^30,31^. Additionally, in a previous study, genetic changes in the early stage of diabetes and the progression of diabetes were studied using partial pancreatectomy in rats^32^, and transcriptomic changes in these results were consistent with our study in terms of reduced expression of Reg family and SNARE genes. Thus, *Crif1*^*beta+/−*^ mice can be considered an early diabetes model.

This ability of β cells to control and modulate their mitochondrial bioenergetics according to nutrient supply is essential to maintain their functionality (nutrient-stimulated insulin secretion) and viability^33^. *Crif1*^*beta+/−*^ mice have partial mitochondrial dysfunction in β cells (Fig. 2i, j). Therefore, we speculate that *Crif1*^*beta+/*−^ mice may have partial bioenergetic insufficiency due to β-cell mitochondrial dysfunction, which may lead to reduced energy expenditure and weight gain^34–36^.

In the present study, it was notable that β-cell-specific *Crif1* haploinsufficiency resulted in the loss of first-phase insulin secretion, with a reduction in the number of docked granules. Many studies have shown that first-phase insulin secretion depends on the availability of membrane docking granules^37–40^. This is important because defects in first-phase insulin secretion have a substantial influence on glucose tolerance^41^. A soluble N-ethylmaleimide-sensitive fusion protein attachment protein receptor (SNARE) protein is responsible for the exocytosis of the granules, and interestingly, β-cell-specific *Crif1* haploinsufficiency was associated with lower expression of the SNARE genes *Stx1*, *Vamp2*, and *Snap25* in islets. Furthermore, consistent with this, it has been reported that low expression of the SNARE proteins STX1, SNAP25, and VAMP2 is associated with insulin secretory defects in rodents and humans with obesity and type 2 diabetes^39,42–44^. First-phase insulin secretion also requires a rapid and marked increase in Ca^2+^ influx, which occurs via the opening of l-type voltage-dependent calcium channels (VDCCs)^45,46^. However, *Crif1* haploinsufficiency was not associated with an impairment in the glucose-stimulated increase in [Ca^2+^]_i_ (Supplementary Fig. 3a), which suggests that the loss of first-phase insulin release in these mice was not the result of impairments in calcium channel opening or calcium influx. Instead, we have shown that *Crif1* haploinsufficiency is associated with a defect in exocytosis that is likely the result of the abnormal expression of SNARE proteins, which causes a defect in first-phase insulin secretion.

The normal glucose tolerance of 22-week-old *Crif1*^*beta+/−*^ mice, despite the blunting of first-phase insulin secretion, can be explained by the higher β-cell proliferation, islet mass and β-cell mass, which compensate for the β-cell mitochondrial dysfunction. We also found higher expression of *Reg* family genes in *Crif1*^*beta+/−*^ mice. The proteins produced by these genes are known to promote proliferation and differentiation and to prevent the apoptosis of cells in various organs^47^. Reg proteins induce cell (trans)differentiation, especially to islet cells, and cell proliferation and Reg expression are age dependent, increase during injury or inflammation and have been reported to be linked to pancreatitis, pancreatic cancer, and diabetes^48–51^. Although *Reg2* knockout is not associated with defects in glucose homeostasis or islet mass in young mice, both aging and HFD-induced obesity are associated with lower islet mass, lower plasma insulin, and glucose intolerance^48^. In addition, Reg3 is secreted during pancreatic inflammation to protect cells from stress^47^. Thus, the higher expression of *Reg* family genes in *Crif1*^*beta+/−*^ mice may be an adaptation aimed at countering the stress induced by the MRP defect in β cells and may explain the higher β-cell proliferation, islet mass, and β-cell mass.

The cellular composition of and cell–cell interactions within islets are crucial for the normal function of adult islets^52,53^. A relative increase in the number of *α* cells in the central core of the islet has been shown in many animal models of β-cell defects, as well as in patients with type 2 diabetes^54,55^. *Crif1*^*beta+/*−^ islets displayed a more centralized distribution of *α* cells, but we did not determine whether this altered distribution of *α* cells is caused by an alteration in β-cell polarity in the presence of an MRP deficiency. Indeed, the increase in the number of central *α* cells in *Crif1*^*beta+/−*^ islets may be a secondary effect of unknown defects in β-cell interactions. Furthermore, an increased number of *α* cells was not associated with a change in the plasma glucagon concentration. Additional studies are needed to investigate the cell–cell interactions.

Taken together, this study demonstrates that mitoribosome competence in β cells is closely related to the functional maintenance of β cells. In addition, heterozygotic *Crif1*^*beta+/−*^ mice have a phenotype that resembles the features of human islets in prediabetes and may represent a new tool for investigating the role of islet pathophysiology in human diabetes patients.

There were some limitations to the present study. First, in the three gene sets that we used in the transcriptomic analysis of human islets, there was heterogeneity in the gene expression because of differences in the participants and assay methods used. Second, analysis of the islets obtained from *Crif1*^*beta+/−*^ mice did not permit the ready identification of β-cell-specific functional effects. Third, the differences in mRNA abundances that were identified using transcriptomic data may not have been accompanied by parallel differences in protein expression. Future studies should aim to define the biological effects of MRP defects in islets using methods, such as single-cell sequencing, proteomics, and tracer studies in reporter mice. Finally, further studies are needed to identify the MRPs that affect the risk of type 2 diabetes in humans.

## Supplementary information

Supplementary

## Data Availability

All data that support the findings of this study are available from the authors on reasonable request. No applicable resources were generated or analyzed during the current study.
