# Supplementary material for: Mitoribosome insufficiency in β cells is associated with type 2 diabetes-like islet failure
Source: Exp Mol Med. 2022 Jul 8;54(7):932–45. doi: 10.1038/s12276-022-00797-x (PMC9355985; doi:10.1038/s12276-022-00797-x)
Supplement: Supplementary file 1 — Supplementary [file 12276_2022_797_MOESM1_ESM.pdf]

Mitoribosome insufficiency in β cells is associated with type 2 diabetes-like islet failure

(Running title: Mitoribosomal regulation and islet function)

Hyun Jung Hong[1,2], Kyong Hye Joung[1,3], Yong Kyung Kim[1], Min Jeong Choi[1], Seul Gi Kang[1], Jung Tae Kim[1,2], Yea Eun Kang[1,3], Joon Young Chang[1,2], Joon Ho Moon[4], Sangmi Jun[5,6], Hyun-Joo Ro[5,6], Yujeong Lee[5,6], Hyeongseok Kim[7], Jae-Hyung Park[8], Baeki E. Kang[9], Yunju Jo[9], Heejung Choi[9], Dongryeol Ryu[9,10,11], Chul-Ho Lee[12], Hail Kim[4], Kyu-Sang Park[13], Hyun Jin Kim[1,3]*, and Minho Shong[1,2,3]*

Content

1. Supplementary material method
2. Supplementary table
3. Supplementary figures

1. Supplementary Material methods

Analysis of differential gene expression using the gene expression omnibus (GEO) databases

Bioinformatic analyses were performed using GSEA (http://www.broadinstitute.org/gsea) and R packages as described previously [1]. The publicly available transcriptomic datasets for human pancreatic β-cells were obtained from the NCBI GEO site under accession numbers GSE25724, GSE76895, GSE76894, and GSE159984. For a meta-analysis of the combined data sets, the expression data from GSE20966, GSE76895, and GSE76894 were $\log_{10}$-transformed [2], and data from GSE76894 and GSE76895 were $\log_{2}$-transformed and normalized, as in a previous study [3]. Then, the pre-processed expression levels were converted into Z-scores [3,4]. All the heatmaps and scatter plots were visualized using RStudio (4.0.2), and the R packages ggplot2, ggpubr, ggpairs, and GGally. For further analyses, raw read count data from the GSE159984 dataset was processed and normalized into transcript per million (TPM), as described previously [5]. The single-cell transcriptome of the human pancreatic islet generated in the previous study [6] was obtained from the European Nucleotide Archive (ENA; accesses number: ERP017126). The gene expression profile of the human pancreatic β-cells was separated. Next, we analyzed and visualized the gene expression on uniform manifold approximation and projection (UMAP) plot with R package M3C and ggplot2 (R version 4.0.3, https://www.r-project.org/).

Islet isolation

A 0.8 mg/ml solution of collagenase P (11213865001, Roche Diagnostics, Mannheim, Germany) was perfused through the bile ducts of the mice, and then the pancreata were removed and placed in 50 ml tubes containing 3 ml collagenase P solution. The tubes were

then incubated in a water bath at 37.5°C for 13 min 20 sec, with the tubes being manually shaken for 2 min during the incubation. The digested solution was centrifuged (230 × g values for 1 min, repeated three times) after filtration through a mesh, and the supernatant was then removed. Fractions were separated using a Ficoll gradient with densities of 1.108, 1.085, 1.069, and 1.037 g/ml (Biocoll separating solution, L6155, Biochrom, Berlin, Germany). The isolated islets were placed in medium 199 (M4530, Sigma Aldrich) and incubated in 5% CO_2 at 37°C.

RNA isolation and quantitative real-time PCR (qPCR)

Islets were isolated as described above, from which RNA was isolated using Trizol Reagent, according to the manufacturer's instructions (15596018, Life Technology, Eugene, OR, USA). cDNA was then prepared using M-MLV reverse transcriptase and oligo-dT primers (28025013, Invitrogen, CA, USA) and used to measure the relative expression of target mRNAs using qPCR. The results were normalized to the expression of Actb and are expressed as fold differences from the control values. The PCR primers used are shown in Supplementary Table 1.

Immunoblot analysis

Western blot analysis was performed on isolated islets. Isolated islets were sonicated (20% for 20 sec) in lysis buffer (50 mmol/l Tris, pH7.4, 150 mmol/l NaCl, 1 mmol/l EDTA, 0.1% Triton X-100) with protease inhibitor (Roche), denatured by boiling for 5 min, and separated by sodium dodecyl sulfate-polyacrylamide gel electrophoresis. The proteins were then transferred to nitrocellulose membrane (10600002, Amersham Bioscience, Germany), which was blocked using Tris-buffered saline (TBS) containing 5% skim milk and 0.1% Tween-20 (Sigma Aldrich) for 20 min. The blots were then incubated with primary antibody diluted in TBS containing 5%

skim milk and 0.01% Tween-20) overnight 4℃. The blots were then washed three times using 1 × TBS containing 0.1% Tween-20 for 10 min each and incubated with peroxidase-conjugated secondary antibodies at a 1:1,000 dilution. Finally, the immunoreactive bands were detected using ECL solution according to the manufacturer's instructions (Advansta, USA). All image were obtained using ODYSSEY instrument and Image Studio^{TM} softwere (LI-COR Biosciences, Lincoln, NE, USA). The antibodies used are shown in Supplementary Table 2.

Intracellular Ca^{2+} measurements

Isolated islets were seeded onto glass coverslips in tissue culture plates, loaded with 1 μmol/l Fura-2 AM in glucose-free DMEM media (11966-025; Gibco), and then stimulated with 11.1 mmol/l glucose. The coverslips were then mounted, and the Fura-2 AM signals of the islets were recorded using an IX-73 inverted microscope platform (Olympus, Tokyo, Japan) attached to a complementary metal-oxide-semiconductor camera (Prime-BSI CMOS camera; Teledyne Photometrics, Tucson, TX, USA) and a LED illuminator (pe-340Fura; CoolLED Ltd., Andover, UK).

The fluorophore was alternately excited at 340 nm and 380 nm, the emission was recorded at 510 nm using MetaFluor6, and then the background-subtracted 340/380 Fura-2 AM ratio, which reflects $[Ca^{2+}]_i$ changes, was calculated. At the end of each experiment, 50 mmol/l KCl in KRB was added to induce a maximal ratio in order to aid comparison between Crif1$^{beta+/+}$ and Crif1$^{beta+/-}$ islets. During the recording of Fura-2 AM signals, the coverslips in the chamber were perfused with Ca^{2+}-free KRB, unless otherwise stated.

RNA-sequencing data processing and analysis

Before RNA sequencing, the quality check was performed through DNA quantity, DNA condition evaluation, and DNA size check, and all were suitable for further analyses. In addition, for each gene, genes with a FPKM value of 0 in at least one of the six samples were excluded from analysis. Thus, statistical analysis was performed on 16,489 genes, excluding 7,966 out of 24,455 genes. Total RNA extracted from these islets (n = 3 each) was sequenced using a NovaSeq 6000 System. The obtained reads were mapped to a reference Mus musculus (mm9) genome using HISAT2 version 2.1.0. The aligned reads were then assembled using known gene/transcript sequences and a reference gene model in StringTie version 1.3.4d. Transcript frequencies were quantified as normalized values, taking into account transcript length and depth of coverage. Relative transcript abundance is expressed as fragments per kilobase of transcript per million fragments mapped (FPKM). One was added to each FPKM value for filtered genes, and the filtered data were $\log_2$-transformed and quantile-normalized. Differentially Expressed Genes (DEG) analysis was performed using the FPKM values.

Metabolic and physiological analyses

For intraperitoneal glucose tolerance testing (IPGTT), mice were fasted for 6 hr, and their blood glucose concentrations were measured before, and 15, 30, 60, and 120 min following the intraperitoneal injection of 1g/kg glucose. Blood glucose levels were measured with a glucometer (GC04640323, Accu-Chek Active, Roche Diagnostics, Mannheim, Germany). For intraperitoneal insulin tolerance testing (IPITT), mice were fasted for 6 hr and then injected intraperitoneally with 0.75 U/kg insulin (VL7510, Humalog, Eli Lilly, Indianapolis, IN, USA). Then, their plasma insulin concentrations were measured using a Mouse Insulin ELISA kit (80-INSMSU-E01, Alpco Diagnostics) in blood collected before, and 15 and 30 min following the insulin injection.

Transmission electron microscopy (TEM)

Isolated islets were pelleted by centrifugation and fixed in a 2.5% solution of glutaraldehyde in phosphate buffer (0.1 M, pH 7.4) for 2 hr at 4°C. The pellets were then stained and embedded in 100% Embed 812 resin for sectioning. Plastic sections of 80 nm thickness were then cut using a Leica EM UC6 ultramicrotome (Leica Microsystems GmbH), and stained with 2% uranyl acetate and 1% lead citrate. The slides were then examined using a Zeiss LEO912AB 120 kV transmission electron microscope (Carl Zeiss) and a FEI Tecnai G2 Spirit Twin 120 kV transmission electron microscope (FEI Company).

Histological analysis

The pancreata were excised, incubated in 10% neutral-buffered formalin (BBC Biochemical, WA, USA) for 6 hr at room temperature, and embedded in paraffin wax for sectioning. Paraffin-embedded sections of 4 μm thickness were cut at 60 μm intervals (total 15 section) and then transferred to polylysine slides (10219280, Thermo Scientific). Four selected sections were stained with hematoxylin and eosin (H&E) or immunofluorescent (IF) or immunohistochemistry (IHC), as previously described [7]. H&E-stained sections were examined using an upright microscope (BX53; Olympus Corp., Waltham, MA, USA), and the areas of the islets were quantified using Imageinside software [7]. Islet mass was calculated as the product of pancreas weight and percentage islet area [8]. For immunofluorescent staining, sections were manually deparaffinized in xylene and rehydrated in ethanol for further staining. Antigen retrieval was performed by incubating the slides in sodium citrate buffer (10 mmol/L sodium citrate, pH 6.0) with a microwave for 15 min. For immunofluorescent staining, samples were blocked with CAS blocker (Thermo) for 2 h at room temperature. Slides treated with the following primary antibodies were incubated at 4°C overnight: anti-Insulin antibody (mouse, dilution factor 1:500; Sigma), anti-Insulin antibody (rabbit, 1:500; CST), anti-Insulin antibody

(guinea pig, 1:200; Invitrogen), anti-glucagon antibody (1:200, mouse; Sigma), anti-ki67 antibody (1:200, rabbit; Abcam), anti-MAFA antibody (1:200, rabbit; CST), and anti-GLUT2 antibody (1:100, Rabbit; Millipore). After incubation and washing, the following secondary antibodies were treated and incubated for 2 h at room temperature: Goat Anti-Rabbit IgG (H+L), Alexa Fluor 568 (1:500; Invitrogen), Goat Anti-Mouse IgG (H+L), Alexa Fluor 488 (1:500; Invitrogen), and Goat Anti-Guinea pig IgG (H+L), Alexa Fluor 647 (1:500; Abcam). Samples were treated with DAPI (1:1,000; Invitrogen) for 1 min and mounted with fluorescence mounting medium (Sigma). IF sections were then examined using an Olympus Fluoview FV1000 microscope equipped with a CCD camera (Olympus Corp., Lake Success, NY, USA), and the β-cell proliferation rate and the numerical ratio of α-to-β-cells in the islets were calculated. The proliferation rate was estimated on the basis of the percentage of cells that were positive for both Ki67 and insulin. For immunohistochemistry staining, the sections were incubated with peroxidase blocker (Thermo) at room temperature for 10 min and blocked with 2% serum and treated with the primary antibodies as above. After washing, biotinylated secondary antibody was incubated with the samples for 1hr at room temperature, and streptavidin (Vector) reaction. Immunohistochemistry was performed using DAB following the manufacturer’s protocol (Thermo). The slides were counterstained with hematoxylin. Immunohistochemistry sections were then examined using an Pannoramic MIDI Ⅱ (3DHISTECH Ltd), and β-Cell mass was calculated as the product of pancreas weight and percentage insulin area [9]. β-cell area was measured by insulin immunoreactive area divided by whole-pancreas area.

References

1 Kim, K. *et al.* Degradation of PHLPP2 by KCTD17, via a Glucagon-Dependent Pathway, Promotes Hepatic Steatosis. *Gastroenterology* **153**, 1568-1580 e1510 (2017).

2 Dickson, W. M. & Rodowskas, C. A., Jr. What are the functions of a practicing pharmacist? *J. Am. Pharm. Assoc.* **16**, 194-199 (1976).

3 Zwiener, I., Frisch, B. & Binder, H. Transforming RNA-Seq data to improve the performance of prognostic gene signatures. *PloS one* **9**, e85150 (2014).

4 Cheadle, C., Vawter, M. P., Freed, W. J. & Becker, K. G. Analysis of microarray data using Z score transformation. *J. Mol. Diagn.* **5**, 73-81 (2003).

5 Hwangbo, H. *et al.* Bio-printing of aligned GelMa-based cell-laden structure for muscle tissue regeneration. *Bioact. Mater.* **8**, 57-70 (2022).

6 Segerstolpe, A. *et al.* Single-Cell Transcriptome Profiling of Human Pancreatic Islets in Health and Type 2 Diabetes. *Cell metab.* **24**, 593-607 (2016).

7 Kim, Y. K. *et al.* Disruption of CR6-interacting factor-1 (CRIF1) in mouse islet beta cells leads to mitochondrial diabetes with progressive beta cell failure. *Diabetologia* **58**, 771-780 (2015).

8 Oropeza, D. *et al.* Phenotypic Characterization of MIP-CreERT1Lphi Mice With Transgene-Driven Islet Expression of Human Growth Hormone. *Diabetes* **64**, 3798-3807 (2015).

9 Ni, Q. *et al.* Raptor regulates functional maturation of murine beta cells. *Nat. Commun.* **8**, 15755 (2017).

2. Supplementary Tables

Supplementary Table 1. Primers used for qRT-PCR

Gene	Forward	Reverse
Crif1	GAA CGC TGG GAG AAA ATT CA	ATA GTT CCT GGA AGC GAG CA
Nd1	ACG CAA AAT CTT AGG GTA CA	GAG TGA TAG GGT AGG TGC AA
Ndufa9	ACT GTG TTT GGG GCT ACA GG	GAT TGA TGA CCA CGT TGC TG
Sdha	ACA CAG ACC TGG TGG AGA CC	GCA CAG TCA GCC TCA TTC AA
Uqcrc2	ATC AAA AGG GGC AAC AAC AC	CAC TCA GGA AGC CCT CTG AC
Cox4	TTG GCA AGA GAG CCA TTT CT	GCC CAC AAC TGT CTT CCA TT
Atp5a	AGG CCT ATC CTG GTG ATG TG	CTT CAT GGT ACC TGC CAC CT
Vamp2	CCC ACA CAC CAG GTT TTC TGT	GCA GGG GAC ACT GGG ATA ATA
Stx1	ATG GAG AAG GCT GAT TCC AAC	CCA TGA GAG AAG CAT GAA GGA
Snap25	GTG AGG AAT TGG AAG ACA TGC	GCC TTG CTC TGG TAC TTG ACG
Reg2	GGG AGG CTG ATC TCT TTT GC	AGG CCA CAA AGT TGC TCT CA
Reg3b	GAA TAT ACC CTC CGC ACG CA	TCT TTT GGC AGG CCA GTT CT
Reg3d	GCA GAA ATG CCA GGT GTC CT	GAA CCA CAG ACC TGG GCT AA
Reg3g	ATG CCC CAT CTT CAC GTA GC	TGG CAG GCC ATA TCT GCA TC
Pcna	ACC TGC AGA GCA TGG ACT CG	GCAGCGGTATGTGTCGAAGC
Ki67	TTG ACC GCT CCT TTA GGT ATG AA	TTC CAA GGG ACT TTC CTG GA
Top2a	AGC AGA TTA GCT TCG TCA ACA GC	ACA TGT CTG CCG CCC TTA GA
Ccnd	GCT ATG GAG CTG CTG TGC T	CCAAGAAACGGTCCAGGTAA
18S	GTA ACC CGT TGA ACC CCA TT	CCA TCC AAT CGG TAG TAG CG
Actb	CAC AGC TTC TTT GCA GCT CCT	GTC ATC CAT GGC GAA CTG G

Supplementary Table 2. Antibodies used for Western blot and Immunofluorescent analysis

**Antibody**	**Source**	**Cat.,RRID**
Rabbit Anti-Ki67	Abcam	Cat#: ab15580; RRID: AB_443209
MAFA (D2Z6N) rabbit monoclonal antibody (Ab)	Cell Signaling Technology (CST)	Cat#: 79737; RRID: AB_2799938
Mouse Anti-glucagon Ab	Sigma Aldrich	Cat#: G2654; RRID: AB_259852
Rabbit Anti-insulin Ab	CST	Cat#: 4590; RRID: AB_659820
Mouse Anti-insulin Ab	Sigma Aldrich	Cat#: I2018; RRID: AB_260137
Guinea pig anti-insulin Ab	Invitrogen	Cat#: PA1-26938; RRID: AB_794668
Rabbit Anti-GLUT2 Ab	Merck Millipore	Cat#: 07-1402; RRID: AB_1587076
Goat Anti-Rabbit IgG (H+L), Alexa Fluor 568	Invitrogen	Cat#: A-11011; RRID: AB_143157
Goat Anti-Mouse IgG (H+L), Alexa Fluor 488	Invitrogen	Cat#: A-11001; RRID: AB_2534069
Goat Anti-Guinea pig IgG (H+L), Alexa Fluor 647	Abcam	Cat#: ab150187; RRID: AB_2827756
Anti-CRIF1 (H-9) Ab	Santa Cruz	Cat#: sc-374122; RRID: AB_10917749
Biotinylated Horse Anti-Mouse IgG	Vector	Cat#: BA-2000; RRID: AB_2313581
Horseradish Peroxidase Streptavidin	Vector	Cat#: SA-5004; RRID: AB_2336509
Anti-NDUFA9 Ab	Abcam	Cat#: ab14713; RRID: AB_301431
Anti-SDHB (FL-280) Ab	Santa Cruz	Cat#: sc25851; RRID: AB_2183458
Anti-UQCRC2 Ab	Abcam	Cat#: ab14745; RRID: AB_2213640
Anti-COX4 (20E8) Ab	Santa Cruz	Cat#: sc-58348; RRID: AB_2229944
Ant-ATP5A1 Ab	Invitrogen	Cat#: 459240; RRID: AB_2532234
Anti-actin Ab	Sigma Aldrich	Cat#: A2066; RRID: AB_476693

3. Supplementary Figures

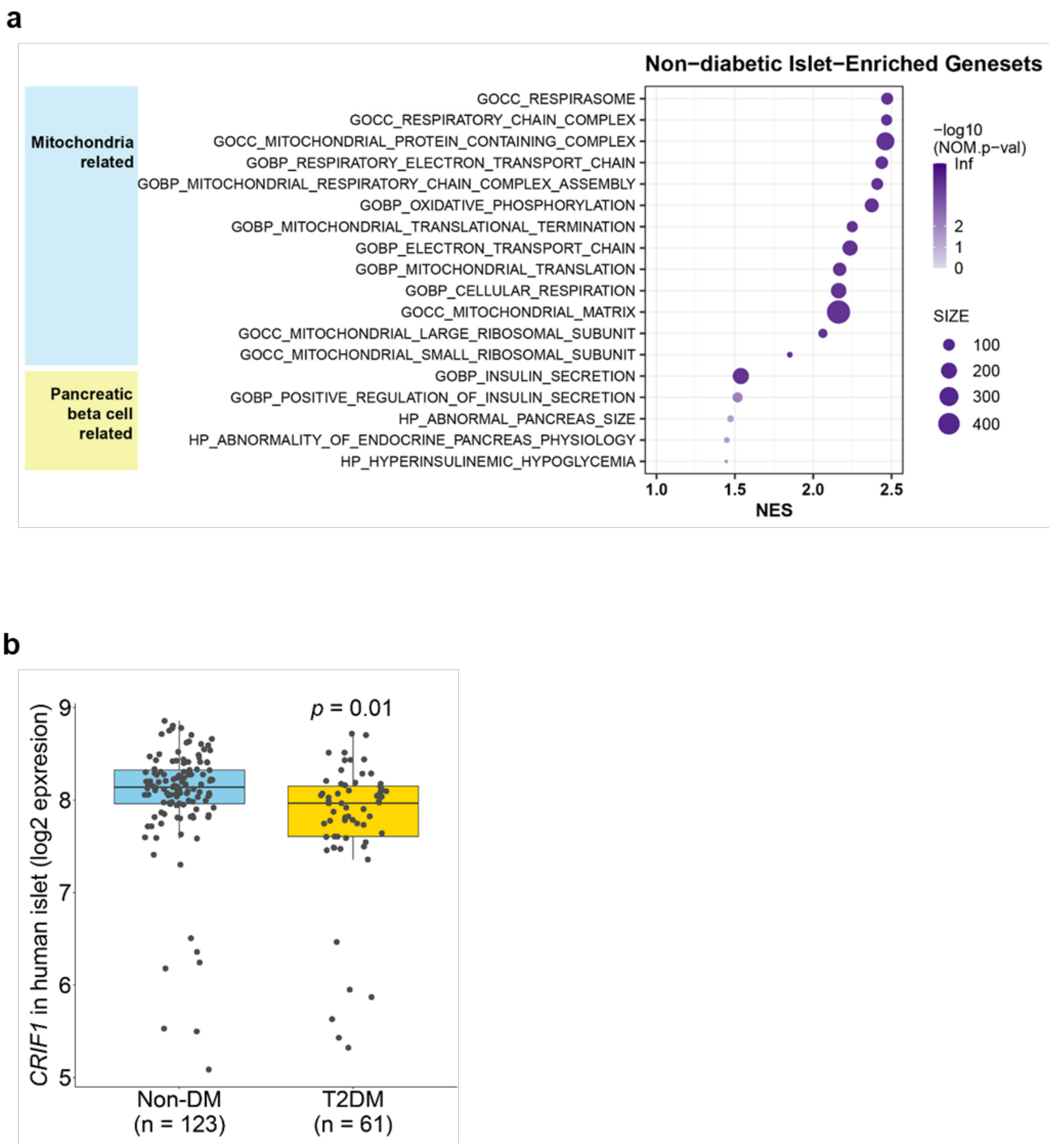

Supplementary Fig. 1 Analysis of human pancreatic islet transcriptomes of type 2 diabetic and non-diabetic organ donors.

(**a**) Bubble plot summarizing the non-diabetic pancreatic islet-enriched gene sets (including all GO term names). (**b**) Boxplot presenting reduced CRIF1 expression in pancreatic islets from donors with type 2 DM compared to the non-DM.

192

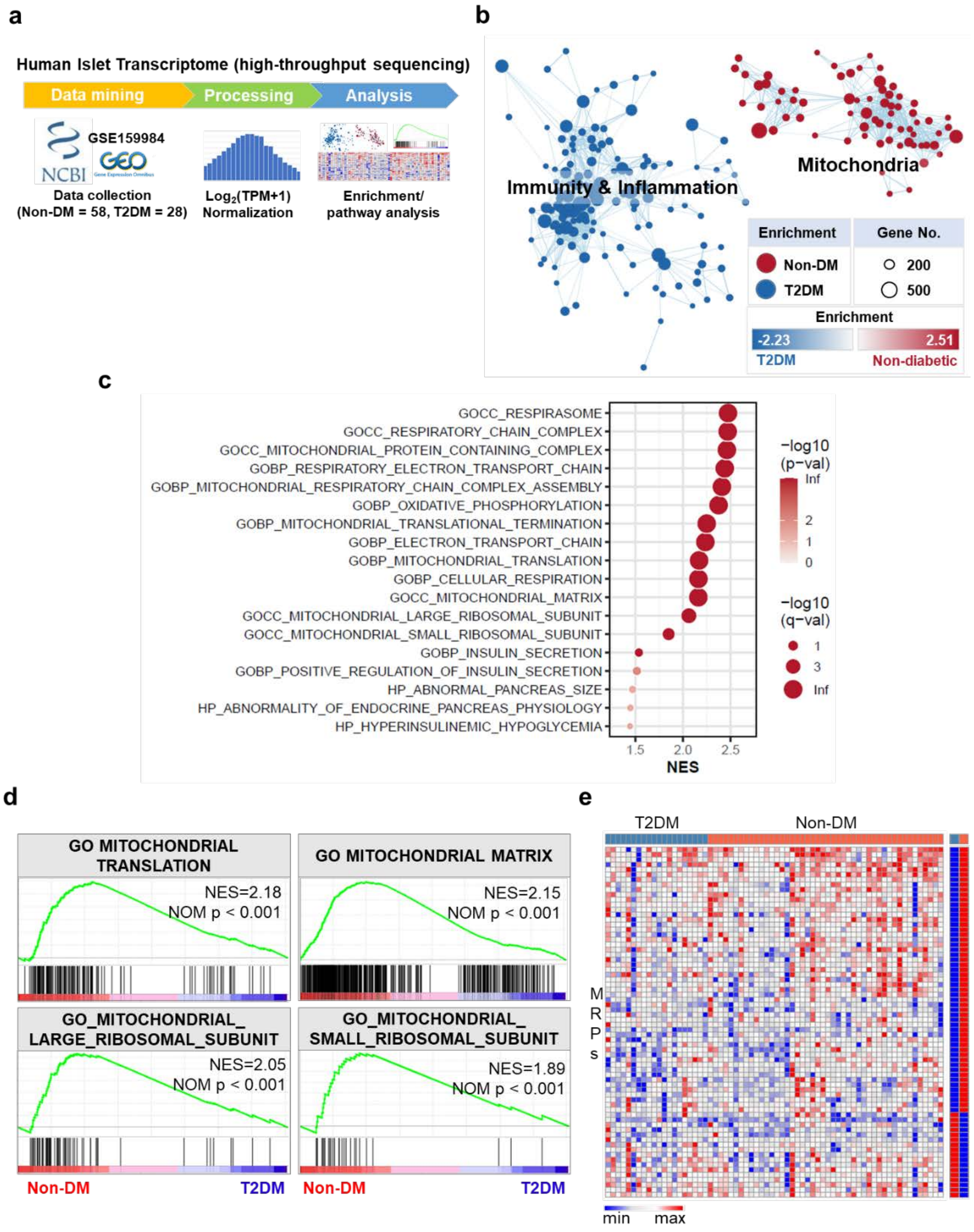
a
Human Islet Transcriptome (high-throughput sequencing)
Data mining
Processing
Analysis
GSE159984
NCBI
GEO
Data collection
(Non-DM = 58, T2DM = 28)
Log2(TPM+1)
Normalization
Enrichment/
pathway analysis
b
Immunity & Inflammation
Mitochondria
Enrichment
Gene No.
Non-DM
T2DM
200
500
Enrichment
-2.23
2.51
T2DM
Non-diabetic
c
GOCC_RESPIRASOME
GOCC_RESPIRATORY_CHAIN_COMPLEX
GOCC_MITOCHONDRIAL_PROTEIN_CONTAINING_COMPLEX
GOBP_RESPIRATORY_ELECTRON_TRANSPORT_CHAIN
GOBP_MITOCHONDRIAL_RESPIRATORY_CHAIN_COMPLEX_ASSEMBLY
GOBP_OXIDATIVE_PHOSPHORYLATION
GOBP_MITOCHONDRIAL_TRANSLATIONAL_TERMINATION
GOBP_ELECTRON_TRANSPORT_CHAIN
GOBP_MITOCHONDRIAL_TRANSLATION
GOBP_CELLULAR_RESPIRATION
GOCC_MITOCHONDRIAL_MATRIX
GOCC_MITOCHONDRIAL_LARGE_RIBOSOMAL_SUBUNIT
GOCC_MITOCHONDRIAL_SMALL_RIBOSOMAL_SUBUNIT
GOBP_INSULIN_SECRETION
GOBP_POSITIVE_REGULATION_OF_INSULIN_SECRETION
HP_ABNORMAL_PANCREAS_SIZE
HP_ABNORMALITY_OF_ENDOCRINE_PANCREAS_PHYSIOLOGY
HP_HYPERINSULINEMIC_HYPOGLYCEMIA
1.5
2.0
2.5
NES
−log10
(p−val)
Inf
2
1
0
−log10
(q−val)
1
3
Inf
d
GO MITOCHONDRIAL
TRANSLATION
NES=2.18
NOM p < 0.001
GO MITOCHONDRIAL MATRIX
NES=2.15
NOM p < 0.001
GO_MITOCHONDRIAL_
LARGE_RIBOSOMAL_SUBUNIT
NES=2.05
NOM p < 0.001
GO_MITOCHONDRIAL_
SMALL_RIBOSOMAL_SUBUNIT
NES=1.89
NOM p < 0.001
Non-DM
T2DM
Non-DM
T2DM
e
T2DM
Non-DM
MRPs
min
max

193

194

Supplementary Fig. 2 Analysis of high-throughput transcriptome sequencing dataset reveals an association between type 2 diabetes and mitoribosomal proteins in human pancreatic islets.

(**a**) Schematic diagram showing the applied analytical workflow. (**b**) Visualization of the enrichment map. Red, gene set enriched in non-diabetic (Non-DM) individuals; blue, gene set enriched in type 2 diabetes (T2DM). The thickness of each line represents the strength of the correlation between nodes, and the size of each circular node represents the size of the gene set. (**c**) Bubble plot showing the normalized enrichment score for each gene set associated with mitochondria and its related functions. (**d**) Enrichment plots for selected gene sets. (**e**) Heatmap showing the MRP gene expression patterns of the GSE159984 dataset.

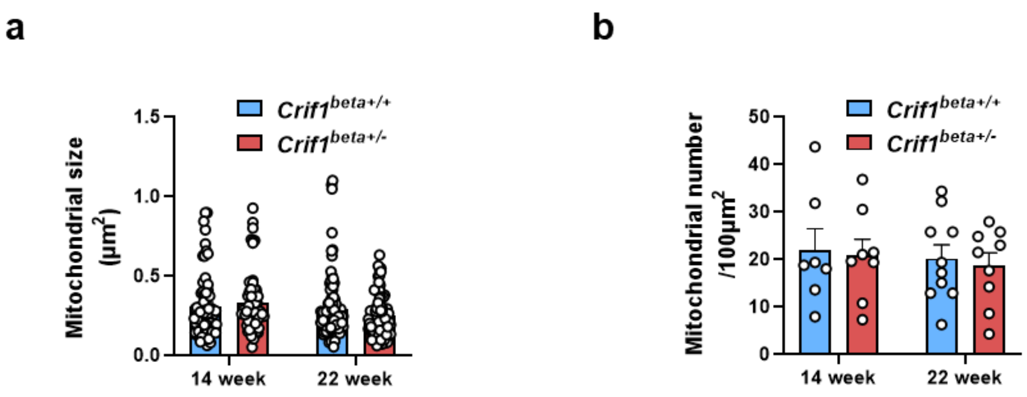

Supplementary Fig.3 *Crif1*$^{beta +/-}$ mice do not change mitochondrial number and size at 14-, 22- week.

(**a**) Mitochondrial size in the β-cell at 14 and 22- weeks of age. (**b**) Mitochondrial number per 100um^2 in the β-cell at 14 and 22- weeks of age

.

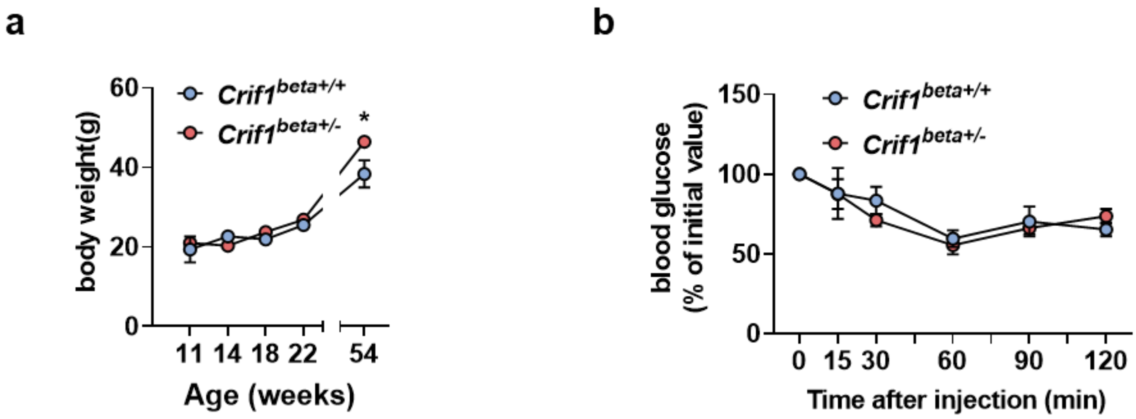

Supplementary Fig.4 *Crif1*$^{beta\ +/-}$ mice show normal body mass and insulin sensitivity until 22-week.

(**a**) Body mass of the mice between 11 and 54 weeks of age; *n* = 3–6. (**b**) Intraperitoneal insulin tolerance testing (IPITT) data after 6 hr of fasting in 22-week-old mice; *n* = 4 per group.

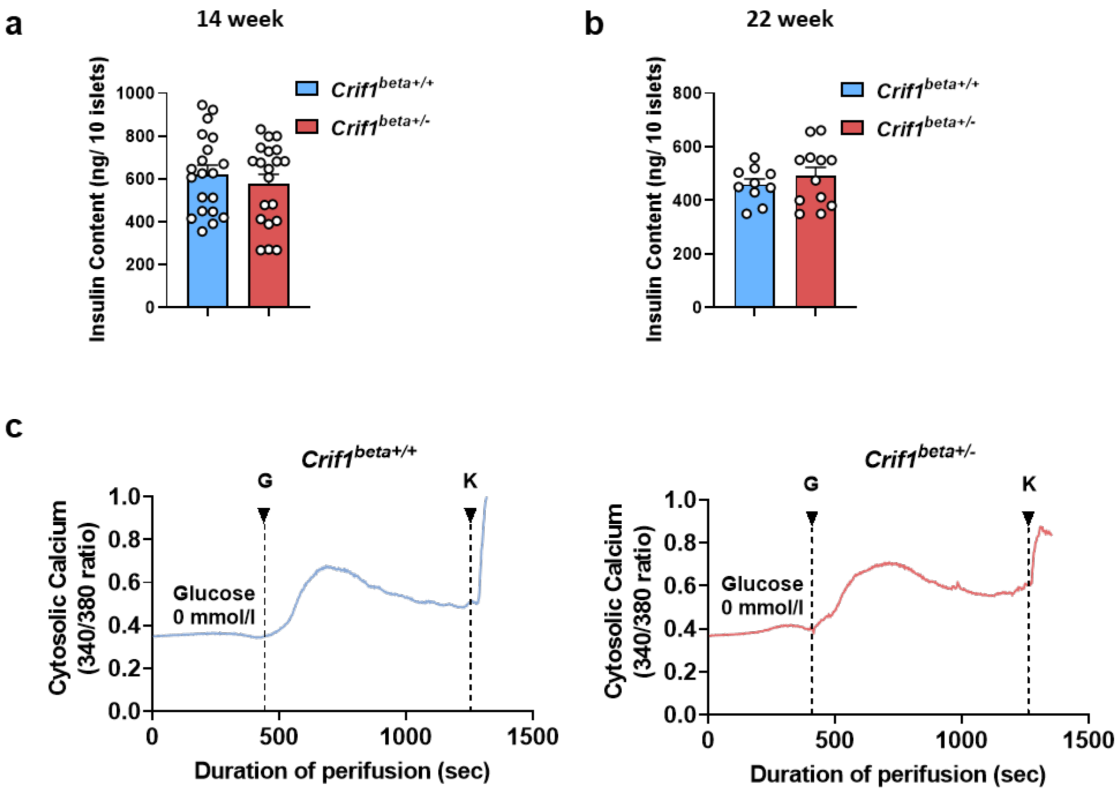

Supplementary Fig.5 insulin content and [Ca^{2+}]$_i$ responses did not differ between *Crif1*$^{beta+/-}$ and *Crif1*$^{beta+/+}$ islets.

(**a,b**) insulin content in 10 islet at 14 and 22- weeks of age (**c**) Time-course of the changes in Fura-2 fluorescence ratio (R 340/380 nm) in single islets. The treatments were 20 mmol/l glucose (G) and 50 mmol/l KCl (K).

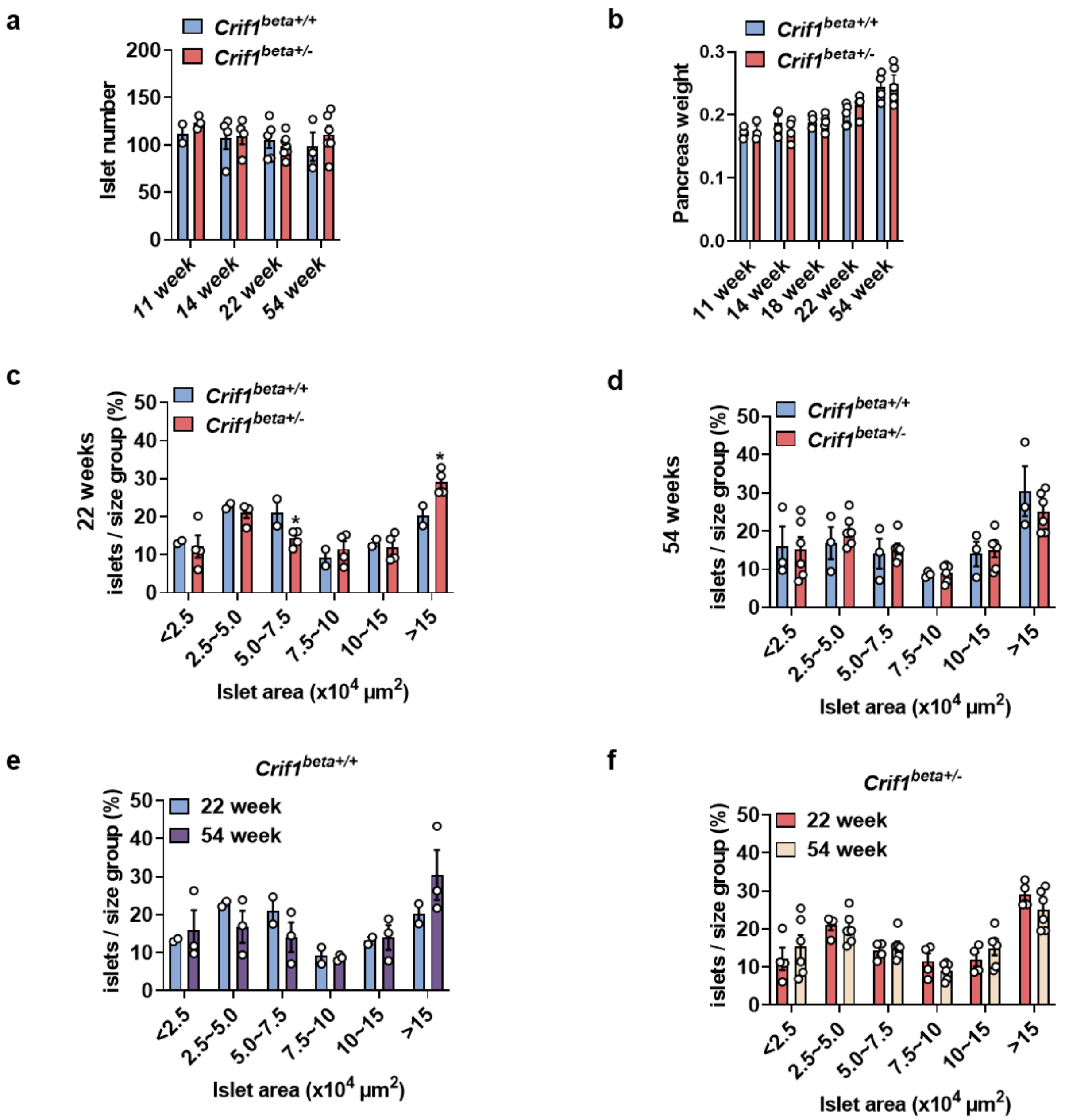

Supplementary Fig. 6 Analysis of islet size distribution of *Crif1*$^{beta+/+}$ and *Crif1*$^{beta+/–}$

(**a**) Total islet number in *Crif1*$^{beta+/+}$ and *Crif1*$^{beta+/–}$ mice of various ages. (**b**) Whole pancreas weight in *Crif1*$^{beta+/+}$ and *Crif1*$^{beta+/–}$ mice of various ages. (**c–d**) The contribution to total islet area of islet size at (**c**) 22 weeks of age, and (**d**) 54 weeks of age. (**e-f**) Comparison of the islet sizes of 22- and 54-week-old (**e**) *Crif1*$^{beta+/+}$ and (**f**) *Crif1*$^{beta+/–}$ mice

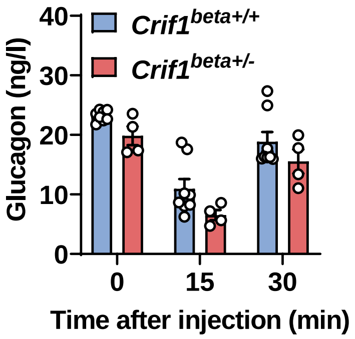

Supplementary Fig.7 Plasma glucagon concentrations in *Crif1*$^{beta+/-}$ mice were similar to those in *Crif1*$^{beta+/+}$ mice

Glucose-stimulated glucagon secretion during intraperitoneal insulin tolerance testing (IPITT) in *Crif1*$^{beta+/+}$ and *Crif1*$^{beta+/-}$ mice (n = 4) at 14 weeks of age.

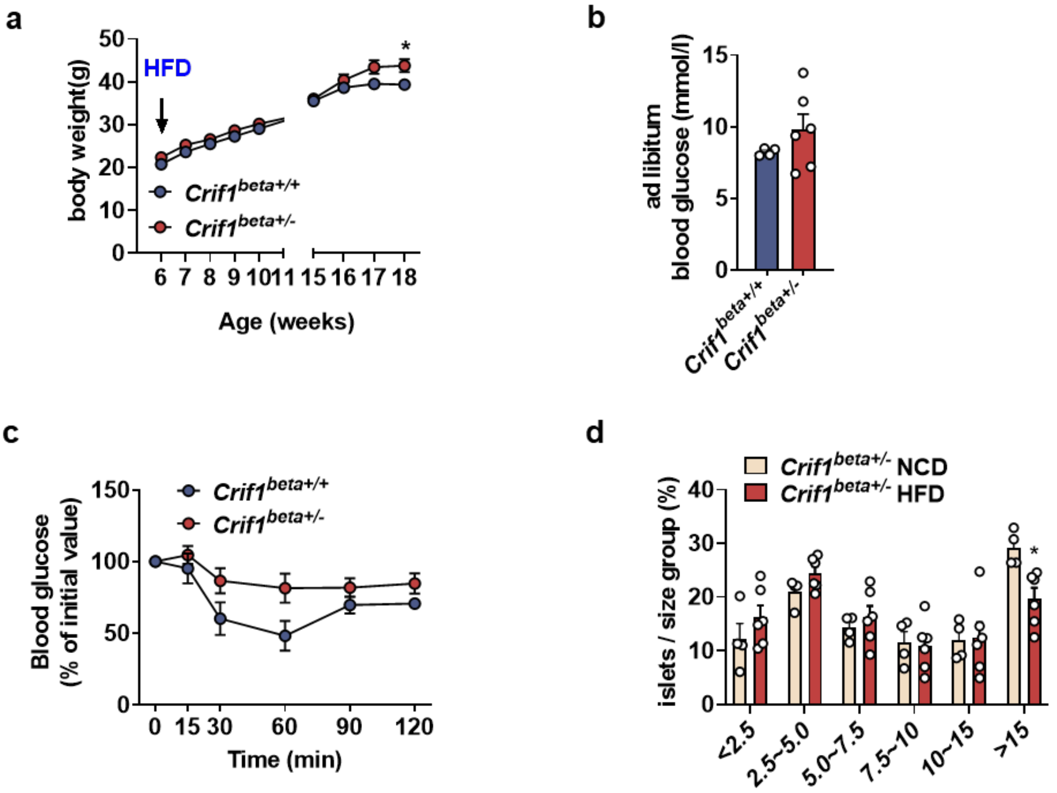

Supplementary Fig. 8 *Crif1*$^{beta+/-}$ mice fail to compensate for high fat diet

(a) Body mass gain after 12 weeks of high-fat diet (HFD)-feeding in *Crif1*$^{beta+/+}$ and *Crif1*$^{beta+/-}$ mice. **(b)** Blood glucose concentrations of *ad-libitum*-fed mice, and **(c)** during intraperitoneal insulin tolerance testing (IPITT) after 6 hr of fasting. **(d)** Contribution to the total islet area of islet size.
